# mCAL: A New Approach for Versatile Multiplex Action of Cas9 Using One sgRNA and Loci Flanked by a Programmed Target Sequence

**DOI:** 10.1534/g3.116.029801

**Published:** 2016-05-13

**Authors:** Gregory C. Finnigan, Jeremy Thorner

**Affiliations:** Division of Biochemistry, Biophysics and Structural Biology, Department of Molecular and Cell Biology, University of California, Berkeley, California 94720-3202

**Keywords:** *Saccharomyces cerevisiae*, CRISPR, essential genes, genome editing, genome engineering

## Abstract

Genome editing exploiting CRISPR/Cas9 has been adopted widely in academia and in the biotechnology industry to manipulate DNA sequences in diverse organisms. Molecular engineering of Cas9 itself and its guide RNA, and the strategies for using them, have increased efficiency, optimized specificity, reduced inappropriate off-target effects, and introduced modifications for performing other functions (transcriptional regulation, high-resolution imaging, protein recruitment, and high-throughput screening). Moreover, Cas9 has the ability to multiplex, *i.e.*, to act at different genomic targets within the same nucleus. Currently, however, introducing concurrent changes at multiple loci involves: (i) identification of appropriate genomic sites, especially the availability of suitable PAM sequences; (ii) the design, construction, and expression of multiple sgRNA directed against those sites; (iii) potential difficulties in altering essential genes; and (iv) lingering concerns about “off-target” effects. We have devised a new approach that circumvents these drawbacks, as we demonstrate here using the yeast *Saccharomyces cerevisiae*. First, any gene(s) of interest are flanked upstream and downstream with a single unique target sequence that does not normally exist in the genome. Thereafter, expression of one sgRNA and cotransformation with appropriate PCR fragments permits concomitant Cas9-mediated alteration of multiple genes (both essential and nonessential). The system we developed also allows for maintenance of the integrated, inducible Cas9-expression cassette or its simultaneous scarless excision. Our scheme—dubbed mCAL for “**M**ultiplexing of **C**as9 at **A**rtificial **L**oci”—can be applied to any organism in which the CRISPR/Cas9 methodology is currently being utilized. In principle, it can be applied to install synthetic sequences into the genome, to generate genomic libraries, and to program strains or cell lines so that they can be conveniently (and repeatedly) manipulated at multiple loci with extremely high efficiency.

Discovery of CRISPR (Clustered Regularly-Interspaced Short Palindromic Repeats)-based RNA-mediated adaptive immunity in bacteria and archaea (Sorek *et al.* 2013; Shmakov *et al.* 2015), and especially the RNA-guided DNA endonuclease Cas9 from the Class II CRISPR system of *Streptococcus pyogenes* (Jinek *et al.* 2012; Doudna and Charpentier 2014), has provided a remarkably versatile tool for modifying genomes (Hsu *et al.* 2014; Wang *et al.* 2016). Combining the normally separate DNA sequence-binding crRNA with the Cas9-stabilizing tracrRNA into a “single-guide” or “synthetic-guide” (sgRNA) streamlined target site recognition (Jinek *et al.* 2012; Ran *et al.* 2013). Changes to the stem-loop architecture of the tracrRNA portion of a sgRNA greatly strengthen its affinity for Cas9 (Chen *et al.* 2013), and shortening of the crRNA portion of a sgRNA to just 20 nucleotides reduces off-target action while preserving efficiency (Pattanayak *et al.* 2013). The range of DNA/chromosome-based applications has been further extended by engineering of *S. pyogenes* Cas9 [or use of Cas9 orthologs from other bacterial species (Jinek *et al.* 2014)] to relax its requirement for initiating DNA sequence recognition at a so-called PAM (“protospacer adjacent motif”) site (5′-NGG-3′) (Kleinstiver *et al.* 2015), to inactivate one or both of its two (McrA/HNH-like and RuvC/RNAaseH-like) catalytic sites to create a “nickase” (Fu *et al.* 2014) or a catalytically “dead” (dCas9) version (Gilbert *et al.* 2013), or to insert new functionalities (Oakes *et al.* 2014). Cas9 and associated sgRNAs have been used in diverse organisms for genome editing, both gene knock-outs (Gaj *et al.* 2013) and gene fusions (Wei *et al.* 2013), as well as to force biased inheritance of a desired allele within entire populations (“gene drives”) (DiCarlo *et al.* 2015; Dong *et al.* 2015; Gantz *et al.* 2015). Cas9-mediated genome alterations have been achieved in bacterial species (Jiang *et al.* 2013; Tsarmpopoulos *et al.* 2016), various fungi (DiCarlo *et al.* 2013; Wagner and Alper 2015), zebrafish (Hwang *et al.* 2013), *Caenorhabditis elegans* (Friedland *et al.* 2013), *Drosophila melanogaster* (Gratz *et al.* 2013), plants (Mao *et al.* 2013), and human cells (Cho *et al.* 2013; Jinek *et al.* 2013; Mali *et al.* 2013; Ran *et al.* 2013), including clinical trials to explore Cas9-mediated therapy in infectious and inherited disease (Kaminski *et al.* 2016; Mendell and Rodino-Klapac 2016; Su *et al.* 2016). Additional applications include sequence-specific repression or activation of gene expression (Cheng *et al.* 2013; Gilbert *et al.* 2013; La Russa and Qi 2015), fluorescent labeling of chromosomal loci (Chen *et al.* 2013, 2016), and RNA-scaffolded recruitment of proteins to a programmed chromosomal localization (Zalatan *et al.* 2015).

For genome editing, the Cas9-sgRNA enzyme allows precise placement of a double-strand break (DSB) at any desired location(s) within a genome of interest. The DSB can be sealed in a highly error-prone manner via nonhomologous end-joining (NHEJ) (Richardson *et al.* 2016; Vriend *et al.* 2016) or, more usefully, by homologous recombination (HR) (typically with PCR products provided in *trans*) to substitute a modification (deletion, insertion, allele replacement, fusion to a reporter sequence, etc.) (Shalem *et al.* 2015; Chandrasegaran and Carroll 2016; Hu *et al.* 2016). Although accuracy and efficiency are generally high, an sgRNA-guided Cas9 can act at other sites in addition to the intended sequence (Cho *et al.* 2014; O’Geen *et al.* 2015; Zhang *et al.* 2015). To reduce such off-target action, specificity-enhancing alterations of Cas9 (Kleinstiver *et al.* 2016; Slaymaker *et al.* 2016) and sgRNA design (Dang *et al.* 2015; Xu *et al.* 2015; Doench *et al.* 2016), and computational methods to search for optimal sgRNA-recognition sites (Bolukbasi *et al.* 2015; Naito *et al.* 2015) have been devised. By the same token, when provided with different sgRNAs concomitantly, Cas9 can effect simultaneous alterations at multiple locations within the genome in any given cell (“multiplex” genome engineering) (Cong *et al.* 2013), and this strategy has been successfully applied in *Saccharomyces cerevisiae*, but almost exclusively to nonessential genes (Ryan and Cate 2014; Bao *et al.* 2015; Horwitz *et al.* 2015; Jakociunas *et al.* 2015; Laughery *et al.* 2015; Mans *et al.* 2015; Ronda *et al.* 2015; Tsai *et al.* 2015). Here, we describe a useful alternative strategy—introduction of unique, programmable, *artificial target sequences* into the genome, thereby permitting multiplex gene manipulation by Cas9 with a single sgRNA.

## Materials and Methods

### Yeast strains and plasmids

All budding yeast strains used in this study can be found in Supplemental Material, Table S1. Standard molecular biology methods were used in this study (Sambrook and Russell 2001). The introduction of the u1 and u2 Cas9 target sites was performed by first cloning vectors using *in vivo* ligation and homologous recombination harboring a single Cas9 site including the PAM sequence (Finnigan and Thorner 2015). As an example, a vector (pGF-V130) containing the 5′ UTR of *CDC11* was digested with a restriction enzyme (*Not*I) downstream of the promoter sequence, and transformed with a PCR fragment of the *CDC11* coding region amplified with oligonucleotides containing overhanging “tails” to insert the Cas9 u1 target sequence in-frame. Two constructs, each with a single flanking u1 site placed upstream or downstream of *CDC11*, were created separately and then combined by a second round of *in vivo* ligation to generate the final construct that contained both flanking u1 sites as well as flanking *CDC11* 5′ and 3′ UTR (330 bp of each). This process was repeated for the *shs1∆*::*Hyg^R^* cassette harboring flanking u1 sites and two Cas9-expressing cassettes containing either u1 or u2 sites at the *HIS3* locus (Table S1). The generated constructs were PCR amplified and integrated into the parent strain in successive yeast transformations. Diagnostic PCRs and Sanger sequencing (Univ. of California, Berkeley Barker Hall Sequencing Facility) of chromosomal DNA were performed to ensure proper integration of all manipulated loci.

Plasmids used in this study can be found in Table S2. Expression of the sgRNA cassettes was modeled after a previous study (DiCarlo *et al.* 2013), using the snoRNA *SNR52* promoter and *SUP4* terminator sequences, and they were synthesized as custom genes with flanking *Xho*I and *Bam*HI restriction sites (GenScript, Piscataway, NJ). The u1 and u2 sequences were chosen from two human genes, SEPT9 and MMP23A, respectively, using the DNA2.0 gRNA Design Tool (DNA2.0, Newark, CA). Putative guide sequences were then examined against the entire yeast genome using a nucleotide BLAST search (National Center for Biotechnology Information) and sequences were considered for having the lowest possible number of matches to the 15 bp sequence (PAM + upstream 12 bp) important for Cas9 “seeding” to minimize off-target effects (Jinek *et al.* 2012; Jiang *et al.* 2013). Additionally, the chosen u1 and u2 sequences were checked against the backbone vector sequences of the pRS316 covering vector, the high-copy sgRNA-expressing pRS425/pRS423 vectors, and both the Kan^R^ and Hyg^R^ cassettes (Goldstein and McCusker 1999), to ensure no highly similar matches existed in these exogenous non-yeast sequences.

### Culture conditions

Yeast were grown in rich YPD or YPGal medium (2% peptone, 1% yeast extract, and 2% dextrose or 2% galactose), or in synthetic medium containing the necessary amino acids with either 2% dextrose or a 2% raffinose and 0.2% sucrose mixture. For transformation of yeast using the Cas9-mediated system, strains were grown overnight in synthetic medium with a raffinose/sucrose mixture lacking uracil (to select for the *CDC11*-expressing WT-covering plasmid) to saturation, back-diluted into YPGal (to an OD_600_ of approximately 0.25–0.35), and grown at 30° for 4.5–5.0 hr. A modified lithium acetate transformation protocol (Eckert-Boulet *et al.* 2012) was used to transform 10 OD_600_ of yeast with combinations of purified plasmid DNA and/or PCR products. Yeast were heat shocked for 45–50 min at 42° and recovered in fresh YPGal overnight at 30° prior to plating onto selective media (selection for both plasmids and no selection for integrated knock-in alleles). An identical transformation protocol was used whether Cas9 was integrated at the *HIS3* locus or expressed on a CEN-plasmid.

The growth of single yeast colonies on various media (G418, Hygromycin, SD-HIS, etc.) was tested by first selecting isolated colonies, creating a small square “patch” (1 cm^2^) on an SD-URA plate, incubating overnight at 30°, and then replica-plating to additional plates to be scored after 1 d of additional incubation. For yeast plates containing a significant number of colonies, the total colony count was estimated in several ways. First, several sectors (half, a quarter, or an eighth, etc.) were selected on the agar plate and the total number of colonies in the sample sector was counted and extrapolated to the entire surface area. Second, subsequent repeated experiments plated various dilutions (1/10, 1/20, 1/50, etc.) of the final transformation product and the total colony counts were added, extrapolated, and averaged together. All experiments were performed in at least triplicate.

### Fluorescence microscopy

For fluorescence microscopy, yeast were grown to saturation overnight in S+Raff/Suc-LEU, back-diluted into YPGal, grown for 5 hr at 30°, harvested, washed with water, and prepared on a standard microscope slide with a coverslip. Samples were immediately imaged on an Olympus BH-2 upright fluorescence microscope (Olympus, Tokyo, Japan) with a 100 × objective lens. A CoolSNAP MYO CCD camera (Photometrics, Tuscon, AZ), a SOLA light source (Lumencore, Beaverton, OR), Micro-Manager software (Edelstein *et al.* 2010), and ImageJ software (National Institutes of Health) were used to process fluorescent images. The cell periphery was determined using an overexposed fluorescence image.

### Polymerase chain reaction and DNA sequencing

All PCR reactions were performed using either high fidelity KOD Hot Start DNA Polymerase (EMD Millipore, Billerica, MA) or PfuUltra II Fusion Hot Start DNA Polymerase (Agilent Technologies, Santa Clara, CA) according to the recommended manufacturer’s conditions (KOD reactions all contained 3 mM Mg^2+^) on a PTC-200 Thermal Cycler (MJ Research, Bio-Rad). Oligonucleotides (Integrated DNA Technologies, Coralville, IA) used in this study can be found in Table S5. For PCR reactions used in the Cas9-mediated integrations, the template DNA was from either purified yeast chromosomal DNA or from bacterial-based plasmids (that cannot be propagated in yeast). Products for integration were confirmed to be the correct size on an agarose gel, but were not purified nor gel extracted; amplified DNA was directly added to the yeast transformation reaction. For diagnostic PCRs to confirm various manipulated loci, DNA agarose gels (1% or 2%) containing Ethidium Bromide were used to separate and image (ChemiDoc System, Bio-Rad Laboratories, Hercules, CA) separated products. Sanger DNA sequencing was performed on all constructed vectors and plasmid intermediates. For sequencing of genomic loci, chromosomal DNA was isolated (Amberg *et al.* 2006) and PCR amplified using a high-fidelity polymerase. The product sizes were confirmed on an agarose gel and the remaining DNA was purified using a QIAquick PCR purification kit (Qiagen, Valencia, CA) and sequenced with overlapping coverage at each desired locus. For diagnostic PCRs, chromosomal DNA was first isolated from yeast strains, as follows. Two precautions were taken to avoid isolating DNA that contained the *URA3*-based covering vector expressing WT *CDC11*. First, cells were grown to saturation under nonselective conditions in rich (YPD) medium overnight (16 hr) at 30°. Second, DNA was isolated using a procedure that recovers only chromosomal DNA (Amberg *et al.* 2006). Indeed, control F2/R2 PCR reactions carried out on DNA isolated using these approaches from the plasmid-containing parental strains GFY-2002 and GFY-2003 demonstrated that the preparations obtained generated PCR products diagnostic for the chromosomal *CDC11* locus, and did not generate any PCR products diagnostic for the plasmid-borne DNA (see Figure 3, Figure S3, and Figure S5).

### Supplemental Material

All supplemental material (Figures S1–S5, Tables S1–S5, and Supplemental References) can be found in File S1.

### Data and reagent availability

We will freely send all plasmids and strains and other research materials and procedures generated from this research to investigators at any and all nonprofit institutions for research purposes upon request.

## Results

### A new strategy for multiplex Cas9-mediated gene editing

When bound to an appropriate sgRNA, Cas9 is able to recognize repeated sequences within a genome, such as telomeres (Chen *et al.* 2013) or the long terminal repeats (LTRs) (δ elements) of the yeast Ty1 retrotransposon (Shi *et al.* 2016). Given that fact, and that current limitations on genome editing by Cas9 include the necessity for an adjacent PAM sequence, the individuality of the desired target sequence itself (to avoid off-target effects), and unknown influences of local chromatin structure, we considered useful ways to circumvent these limitations.

In brief, we first integrate, both upstream and downstream of any locus of interest, a unique 23-nucleotide sequence (a 20 bp target sequence plus a PAM) that has no detectable counterpart in the genome of interest. Second, we integrate, at a safe harbor locus, a cassette that expresses from an inducible Pol II promoter *S. pyogenes* Cas9 bearing a potent universal nuclear localization signal (NLS), which is also flanked by the same or a different unique 23-nucleotide sequence. Third, introduction by DNA-mediated transformation of a plasmid that expresses, from a Pol III promoter, a single sgRNA that matches the unique 23 bp target, along with PCR fragments to replace the excised loci by HR, completes the system.

**Figure 1:**
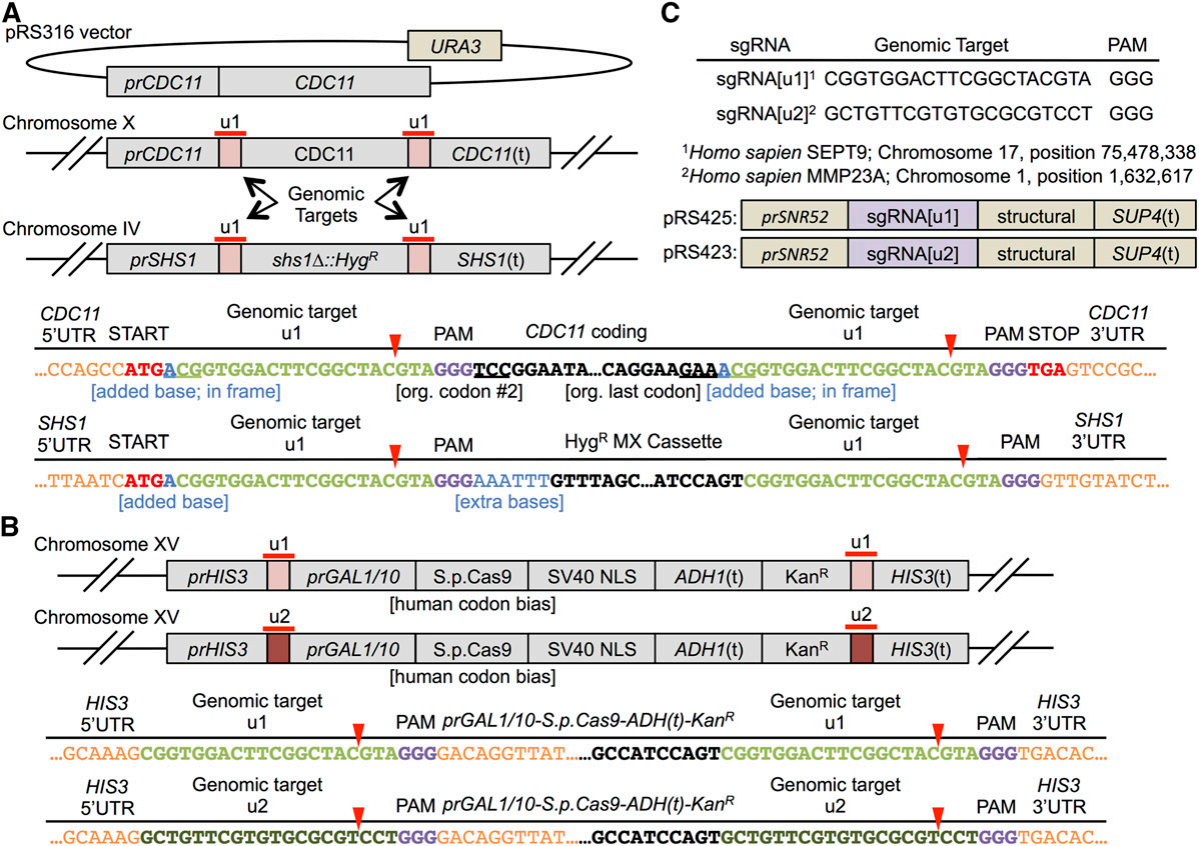
Installation of programmed non-yeast Cas9 target sites at multiple loci. (A) Haploid yeast strains were constructed in which the endogenous *CDC11* gene and a *shs1∆*::*Hyg^R^* allele were flanked by an identical 23 bp sequence containing a Cas9 target site (including a 5′-NGG-3′ PAM sequence) from the human *SEPT9* gene, designated “unique Cas9 site 1,” u1. At *CDC11*, the upstream u1 site was placed in-frame with the initiator Met of the ORF, and the downstream u1 was kept in-frame with the stop codon (via addition of an A to the 5′-end of each u1). Because *CDC11* is an essential gene, a *URA3*-marked *CEN* plasmid expressing WT *CDC11* (but with no 3′-UTR) was also present. *Red triangles*, site of Cas9-directed DSB (+ 3 upstream of the PAM). (B) A cassette for inducible *GAL1/10* promoter-driven expression of S.p.Cas9 bearing a C-terminal SV40 NLS and a *ADH1* transcriptional terminator was used to replace the ORF at the endogenous *HIS3* locus. In one variant (strain GFY-2002), this cassette was flanked by u2, a different 23 bp human sequence containing a Cas9 target from the human *MMP23A* locus. In another variant (strain GFY-2003), the cassette was flanked by u1. (C) The corresponding sgRNA[u1] and sgRNA[u2] sequences were expressed using the constitutive yeast pol III snoRNA *SNR52* promoter and yeast pol III tRNA *SUP4* terminator on high-copy (2 μm DNA) plasmids. DSB, double-strand break; Hyg^R^, hygromycin resistance; Kan^R^, kanamycin resistance; NLS, nuclear localization signal; ORF, open reading frame; PAM, protospacer adjacent motif; sgRNA, single-guide RNA; tRNA, transfer RNA; UTR, untranslated region; WT, wild-type.

As proof of principle, we chose genes encoding two members of the family of mitotically-expressed septins, *CDC11* and *SHS1*, to illustrate the utility of our method for exploiting the features of Cas9-mediated gene manipulation. *CDC11* is an essential gene, whereas cells lacking *SHS1*, although not normal, are viable (Hartwell 1971; Iwase *et al.* 2007; Garcia *et al.* 2011; McMurray *et al.* 2011; Finnigan *et al.* 2015). At the genomic loci for both *CDC11* (Chromosome X) and an *shs1∆*::*Hyg^R^* allele (Chromosome IV), we used standard techniques to insert (see *Materials and Methods*), both upstream and downstream of these two ORFs, a human, 23 bp (or 24 bps if necessary to maintain the reading frame) PAM-containing sequence (designated “u1”), which does not match any other site in the *S. cerevisiae* genome by more than a few nucleotides (Figure 1A). To flank the genes of interest with the u1 (or u2) sequence, two successive rounds of *in vivo* homologous recombination-mediated plasmid assembly in yeast (Finnigan and Thorner 2015) were used to separately introduce these target sites at each end of the desired genes. The resulting constructs were then PCR-amplified and used to transplace the endogenous chromosomal locus of interest by integrative recombination, as described in *Materials and Methods*. Of course, alternative methods could be used to insert the same (or other) Cas9 target sequences upstream and downstream of a gene of interest, including *in vitro* Gibson cloning (Gibson 2011), inverse PCR with extended oligonucleotide tails (Hartl and Ochman 1996), or artificial gene synthesis (Stemmer *et al.* 1995). Moreover, “traditional” Cas9-introduced double-strand breaks (Jinek *et al.* 2012) and appropriate PCR products for their repair could be used to introduce unique sites at desired locations in the genome. The directed placement of the u1 and/or u2 sites can be at any position flanking or within a gene (its UTR sequences, coding sequence, or introns). In our test cases, we inserted the u1 motifs as part of the coding sequence of one gene of interest (*CDC11*) and flanking a Hyg^R^-marked deletion of another (*SHS1*). The former resulted in an eight-residue insertion at the N-terminal end and an eight-residue insertion at the C-terminal end of the Cdc11 polypeptide. Complementation tests revealed that, at least for Cdc11, such small N- and C-terminal extensions are tolerated *in vivo* (data not shown).

Such short “foreign” sequences are far below the length necessary for spontaneous loop-out from a yeast chromosome by HR, as observed, for example, with introduced *Salmonella hisG* repeats (1100 bp) (Alani *et al.* 1987) or the LTRs of retrotransposons (323–424 bp) (Neuveglise *et al.* 2002). In the same strain (Table S1), a cassette expressing *S.p*.Cas9 bearing an SV40 NLS (DiCarlo *et al.* 2013) under control of the inducible *GAL1/10* promoter was integrated at the *HIS3* locus marked by a Kan^R^ gene (Chromosome XV), flanked by u1 or by a different (u2) unique 23 bp PAM-containing sequence (Figure 1B). To demonstrate how this method can be used to replace essential genes with a desired construct, the strain also contained a “covering” plasmid carrying WT *CDC11* and *URA3* (a marker that can be counterselected on 5-FOA medium) (Boeke *et al.* 1984) (Figure 1A). To initiate genome editing, a 2 μm DNA plasmid expressing sgRNA[u1] (Figure 1C) and PCR fragments to integrate at each locus are introduced by transformation into cells in which Cas9 expression has been induced.

The rationale for flanking the target genes with identical sites for Cas9-catalyzed DSB formation is to demand repair of the resulting chromosomal lesions by HR with the PCR fragments provided, permit concurrent replacement of multiple loci using just a single sgRNA, allow for concomitant self-excision of the Cas9-expressing cassette, when desired, and avoid the spurious events that can occur upon standard multiplex Cas9 genome editing (see Figure S1).

### Multiplexing Cas9 to a programmed genomic target sequence using a single sgRNA

**Figure 2:**
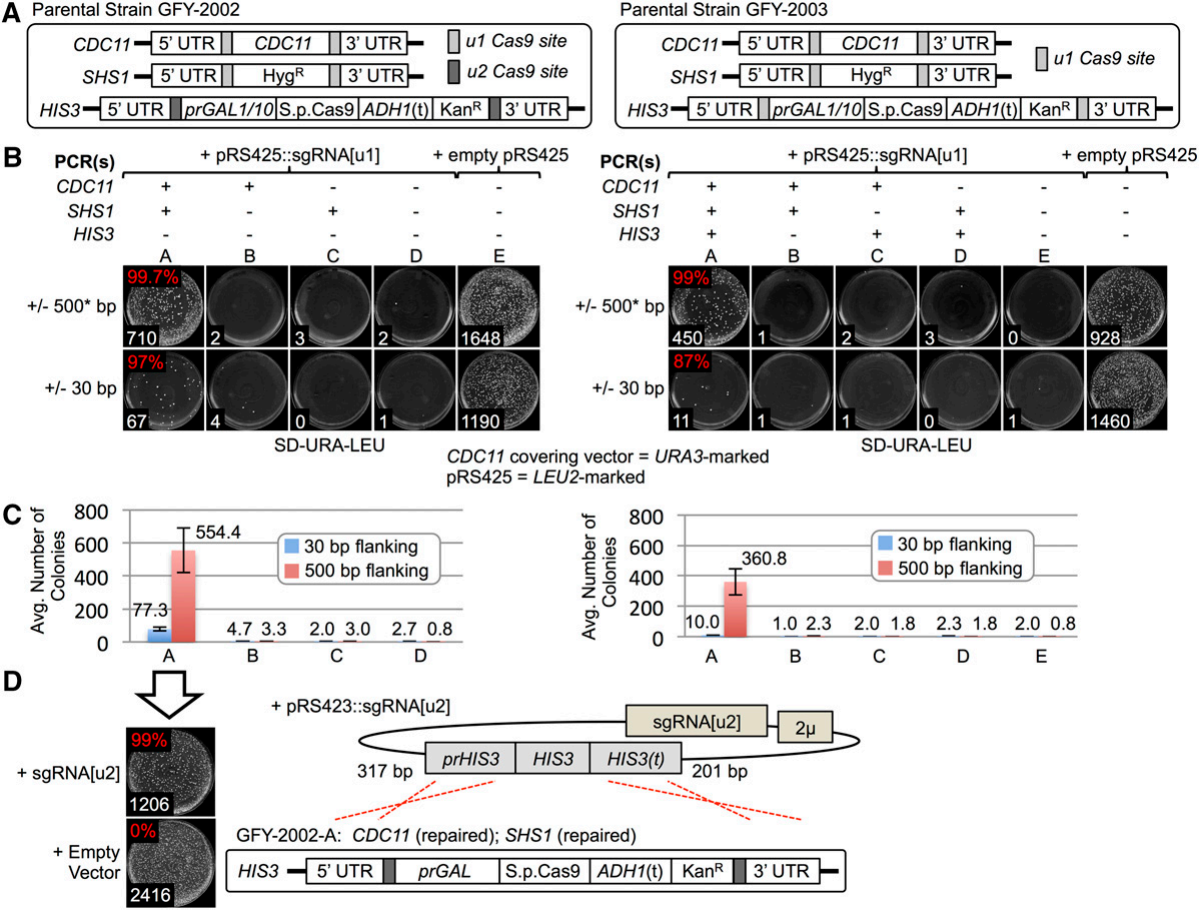
Multiplex Cas9-mediated scarless gene replacement (including an essential gene) and optional concurrent elimination of Cas9. (A) Otherwise isogenic yeast strains containing six programmed Cas9 target sites. In strain GFY-2002, the *CDC11* and *shs1∆*::*Hyg^R^* loci are flanked by u1, whereas the Cas9 expression cassette at the *HIS3* locus is flanked by u2. In strain GFY-2003, all three loci are flanked by u1. Both strains also carried a *URA3*-marked *CEN* plasmid harboring WT *CDC11*. (B) Cas9 expression was induced in strains GFY-2002 (left) or GFY-2003 (right), and then the cells were transformed with an empty *LEU2*-marked vector (pRS425) or with the same plasmid expressing sgRNA[u1] in the absence or presence of various combinations of PCR fragments that span each of the genomic loci of interest, as indicated. The PCR fragments contained either 500 bp (upper plates) or just 30 bp (lower plates) of homology to the genomic sequence flanking each locus. Asterisk, for the *CDC11* PCR fragment, the flanking homology was 330 bp. After recovery in rich medium containing galactose (to support continued Cas9 expression), the cells were plated on SD-Ura-Leu medium. The plates were imaged and the number of colonies recovered were counted after incubation at 30° for 3 d. Each independent trial was performed in triplicate. Representative plates are shown; white numbers, total colony count. The empty vector control confirmed that these conditions allowed for efficient transformation and selection for the *LEU2*- and *URA3*-marked plasmids. Individual colonies from Condition A, where all of the PCR fragments necessary to heal the Cas9-sgRNA[u1]-generated DSBs were provided, were tested for growth on various diagnostic media to ascertain whether successful gene replacement occurred (see Table S3). Red values, percentage of colonies scored that exhibited successful gene replacement at all loci tested. (C) The average colony count over all experimental trials for each condition (A–D), as indicated. Error bars, SEM. (D) An isolate of GFY-2002 from Condition A (B and C) in which both the u1-flanked *CDC11* locus and u1-flanked *shs1∆*::*Hyg^R^* allele were successfully replaced with WT *CDC11* (see Figure 3) and WT *SHS1*, respectively, was grown in galactose to induce Cas9 expression, and then transformed with either empty vector (pRS423) or the same plasmid expressing sgRNA[u2], plated on SD-Ura-His medium, and grown at 30° for 3 d. The selectable marker in the sgRNA[u2]-expressing plasmid is the *S. cerevisiae HIS3* gene with 317 bp of 5′- and 201 bp of 3′-flanking genomic sequence. Therefore, this plasmid not only provides sgRNA[u2] to target Cas9 cleavage at the u2 sites flanking the *his3∆*::Cas9::Kan^R^ cassette, but it also serves as a source of WT *HIS3* DNA to repair the cleaved locus. Representative plates are shown; white numbers, total colony count. To assess conversion of the u2-flanked *his3∆*::Cas9::Kan^R^ cassette to WT *HIS3*, the His^+^ Ura^+^ colonies obtained were scored for loss of G418 resistance and complete elimination of the entire cassette (Table S4). Red values, percentage of colonies scored that exhibited successful elimination of the *his3∆*::Cas9::Kan^R^ cassette. Hyg^R^, hygromycin resistance; Kan^R^, kanamycin resistance; ORF, open reading frame; PCR, polymerase chain reaction; sgRNA, single-guide RNA; UTR, untranslated region; WT, wild-type.

We confirmed, first, that Cas9 is expressed in a galactose-inducible manner and properly localized to the nucleus (Figure S2A) and, second, that expression of neither Cas9 alone, nor sgRNA[u1] or sgRNA[u2] alone (Table S2), nor coexpression of Cas9 with either guide RNA, in otherwise WT cells (*i.e.*, lacking u1 or u2 sequences), caused any detectable loss of viability or transformation efficiency (Figure S2B). We constructed two tester strains, one (GFY-2002) for simultaneous manipulation of two loci (*CDC11* and *shs1∆*::*Hyg^R^* loci) (Figure 2A, left*)* and one (GFY-2003) for simultaneous manipulation of three loci (*CDC11*, *shs1∆*::*Hyg^R^*, and *his3∆*::Cas9::Kan^R^) (Figure 2A, right). After induction of Cas9, these strains were transformed with either empty vector or the same plasmid expressing sgRNA[u1] in the absence or presence of PCR fragments bearing homology to the genomic sequence upstream and downstream of each locus (Figure 2B, upper). Additional control reactions were conducted in the absence of Cas9 expression or in the absence of sgRNA[u1] (Table S3). The PCR fragments used contained either 500 or 30 bp of flanking genomic sequence homology (Figure 2B, lower). Control strains, in which Cas9 cleavage at the u1 sites produces DSBs that have no corresponding PCR fragment(s) for their repair, yielded very few viable colonies [Figure 2, B and C (conditions B–D in figure part B)], even though the intrinsic transformability of the cells was robust (Figure 2B, condition E), because Cas9-mediated DSB formation (with no subsequent repair of the locus) is lethal in yeast. By contrast, we observed a ≥ 300-fold increase in the recovery of viable colonies when the PCR fragments present to mediate repair by HR had 500 bp of homology to the genomic sequence flanking each locus, and a 20–40-fold increase in recovery of viable colonies even when the homology was only 30 bp [Figure 2, B and C (condition A in figure part B)]. For the GFY-2002 strain (Figure 2, left), the viable colonies recovered correspond to successful HR-mediated repair of two loci (*CDC11* and *SHS1*), and for strain GFY-2003 (Figure 2, right), the viable colonies recovered correspond to simultaneous successful repair at all three loci (*CDC11*, *SHS1*, and *HIS3*). Phenotypic characterization showed that, as expected, the vast majority (≥ 97%) of ∼200 randomly-chosen GFY-2002 survivors had become hygromycin-sensitive, and the vast majority (≥ 87%) of ∼200 randomly-chosen GFY-2003 survivors had become both hygromycin-sensitive and G418-sensitive (the status of *CDC11* had to be scored by other means; see below).

Unlike strain GFY-2003, where removal and replacement of the u1-flanked Cas9-expressing cassette occurs concomitantly with multiplex substitution at the other u1-flanked loci, strain GFY-2002 contains a Cas9 expression cassette flanked by u2, a different unique target site. This arrangement allows for additional Cas9-dependent integration (or deletion) events at other loci, if desired, but also allows for excision of the *his3∆*::Cas9::Kan^R^ cassette upon introduction of a plasmid expressing sgRNA[u2]. To test the efficacy of this sequential scheme for removal of Cas9, an isolate of GFY-2002, in which direct DNA sequence analysis showed that sgRNA[u1]-driven genome editing had resulted in restoration of WT *CDC11* and *SHS1* at both loci (Figure 2, B and C, condition A), was transformed with an empty *HIS3*-marked vector (pRS423) or a derivative-expressing sgRNA[u2] cassette (Table S2). In this plasmid, the *HIS3* gene is flanked with significant lengths of genomic sequence (Figure 2D, right); therefore, in theory, it serves both as the source of sgRNA[u2] to catalyze Cas9-mediated excision of the Cas9-expressing cassette and the source of the homologous DNA needed to repair the cleaved locus, without the necessity of cotransforming any PCR fragment or oligonucleotide. Indeed, reassuringly, nearly all (99%) of ∼200 His^+^ colonies obtained from cells exposed to sgRNA[u2] were G418-sensitive, indicating loss of the Cas9-expressing cassette, whereas all of ∼200 His^+^ colonies exposed to the empty vector were Kan^R^, as expected for retention of the Cas9-expressing cassette (Figure 2D, left). The 2 μm DNA-based plasmids used to express sgRNA[u1] or sgRNA[u2] are themselves rapidly lost when not subjected to selection for the appropriate marker (Table S4).

### Confirmation of successful multiplex gene replacement

**Figure 3:**
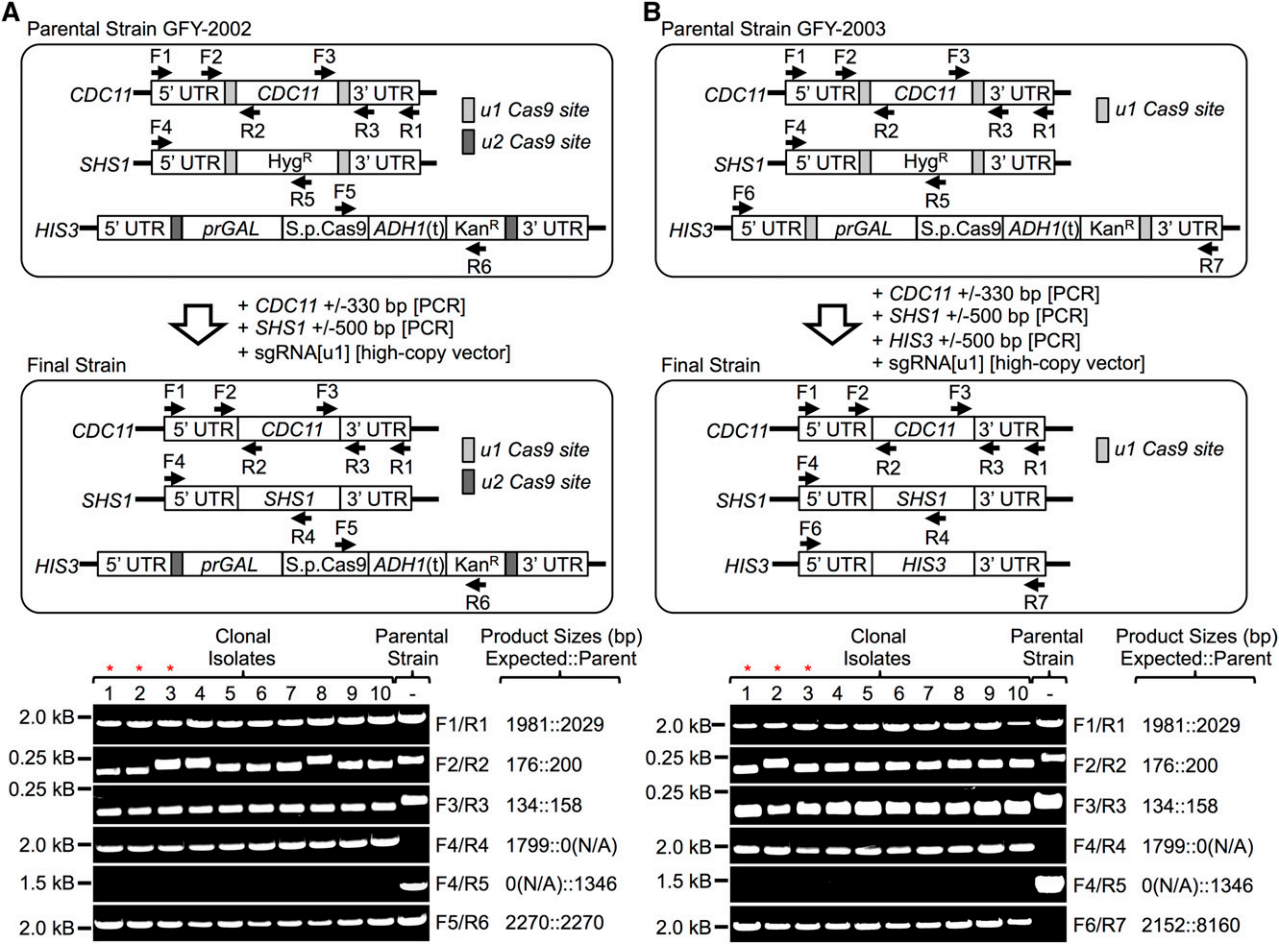
Diagnostic PCR confirms efficient multiplex gene replacement. (A) Chromosomal DNA was purified (Amberg *et al.* 2006) from 10, randomly chosen, clonal isolates from transformations of GFY-2002 in which PCR fragments with 500 bp of flanking genomic homology were provided to restore WT *CDC11* and WT *SHS1* loci, and which had lost the Hyg^R^ marker (see Figure 2B), and tested by PCR with the indicated diagnostic primer sets. An identical analysis was performed on 10 isolates in which PCR fragments with only 30 bp of flanking genomic homology were provided and which had lost the Hyg^R^ marker (see Figure S3). The PCR products were resolved by agarose gel electrophoresis and visualized by staining with ethidium bromide. For *CDC11* (top three gels), the entire locus was amplified (primers F1/R1), as well as small fragments flanking the upstream (F2/R2) or downstream (F3/R3) u1 sites to determine whether or not the Cas9 target site was still present. For *SHS1* (fourth and fifth gels), PCR was performed using primers unique to either *SHS1* itself (F4/R4) or to the Hyg^R^ cassette (F4/R5). Finally, the *HIS3* locus (bottom gel) was testing using a unique primer internal to the Cas9 gene and to the Kan^R^ cassette (F5/R6). For optimal separation, 2% agarose was used for the second and third gels, 1% agarose was used for all of the others. Left, nearest DNA size marker (in kb) for each independent gel; right, expected PCR product sizes. (B) The same kind of analysis as in (A) was performed on chromosomal DNA purified from 10, randomly chosen, clonal isolates from transformations of GFY-2003, except that, in addition, PCR diagnostic for the *HIS3* locus was performed (F6/R7) to ascertain whether the u1-flanked *his3∆*::Cas9::Kan^R^ cassette had been replaced by the WT *HIS3* gene. Left red asterisks, three representative isolates of GFY-2002 that diagnostic PCR indicated carried WT *CDC11* and WT *SHS1* loci, and retained the u2-flanked *his3∆*::Cas9::Kan^R^ cassette, were confirmed as such by direct DNA sequencing (data not shown). Right red asterisks, three representative isolates of GFY-2003 that diagnostic PCR indicated carried WT *CDC11*, WT *SHS1*, and WT *HIS3* loci, were also confirmed as such by direct DNA sequencing (data not shown) [for diagnostic PCR and sequencing of surviving colonies from controls (Figure 2B, conditions B–D), see Figure S5]. Hyg^R^, hygromycin resistance; Kan^R^, kanamycin resistance; ORF, open reading frame; PCR, polymerase chain reaction; sgRNA, single-guide RNA; UTR, untranslated region; WT, wild-type.

Genomic DNA from 10 randomly-chosen colonies from transformations with PCR fragments containing 500 bp of homology (Figure 2, condition A) was analyzed by diagnostic PCR (Figure 3) and direct nucleotide sequencing (data not shown) to examine each manipulated locus. Diagnostic PCR was also performed on colonies from transformations with PCR fragments containing only 30 bp of homology (Figure 2, condition A) with very similar results (Figure S3). For GFY-2002, PCR analysis showed that all 10 isolates replaced the *shs1∆*::*Hyg^R^* allele with the WT *SHS1* gene (and, as expected, still harbored the Cas9-expression cassette), and seven of 10 also properly replaced the u1-flanked *CDC11* locus with the WT *CDC11* gene, which was further confirmed by sequencing. Multiple PCRs tested for the presence or absence of the upstream and downstream u1 sites present at the *CDC11* locus; DNA of the covering plasmid expressing WT *CDC11* was not present, since amplification of the parental strains (control lanes) only displayed single PCR bands corresponding to the chromosomal locus (Figure 3 and Figure S3).

For GFY-2003, all 10 isolates replaced the *shs1∆*::*Hyg^R^* allele with the WT *SHS1* gene and also replaced the Cas9-expression cassette with the WT *HIS3*. For both, the PCR fragments used for gene replacement shared homology only with the genomic sequences flanking these two loci. In the same 10 isolates, nine also properly replaced the u1-flanked *CDC11* locus with the WT *CDC11* gene and, in the remaining one, only the upstream u1 site was retained. In 36 total isolates tested from all experimental trials, 32 replaced both the upstream and downstream u1 sites with WT *CDC11* and only four retained just the upstream u1 site. The most likely explanation for these few exceptions arises from the fact that the *CDC11*-containing PCR fragment we used for replacement shares homology across its entire coding region with the u1-flanked chromosomal *CDC11* locus, and that the upstream u1 site lies just downstream and in-frame with the Met codon need to initiate Cdc11 translation. Thus, crossovers between the PCR fragment and the chromosome that occur within the *CDC11* ORF and in the 3′-UTR will heal a DSB at the downstream u1 site and yield a viable cell that can produce Cdc11, yet retain the upstream u1 (Figure S4). These rare exceptions can be readily avoided by eliminating the internal homology by (i) starting with a genomic *cdc11∆* null allele (covered by WT *CDC11* on a plasmid) or (ii) installing in the chromosome a synthetic ORF with codon alterations that minimize its nucleotide sequence identity to the authentic *CDC11* ORF on the PCR fragment.

Prior work has shown that repair of a DSB via HR in yeast is orders of magnitude more frequent than by NHEJ (Storici *et al.* 2003), which is extremely inefficient (Rattray *et al.* 2001; Daley *et al.* 2005; Storici *et al.* 2006). Indeed, in 16 of the very rare survivors obtained from the controls where the transformations lacked one or more PCR fragments to repair the DSBs (Figure 2, conditions B–D), phenotypic analysis (Table S4) and diagnostic PCR (Figure S5) showed that the majority did not have any replacements and likely escaped any Cas9-induced DSBs and a few isolates replaced one locus, but failed to cut and remove the u1 sites elsewhere. In only three (from Figure 2, condition B), the *shs1∆*::*Hyg^R^* allele was excised, but left a single intact u1 site, most consistent with repair of the Cas9-induced DSBs via HR between the u1 repeats rather than by NHEJ. We conclude from our data that correct replacement at all loci examined is nearly three orders of magnitude more frequent than any other event.

## Discussion

**Figure 4:**
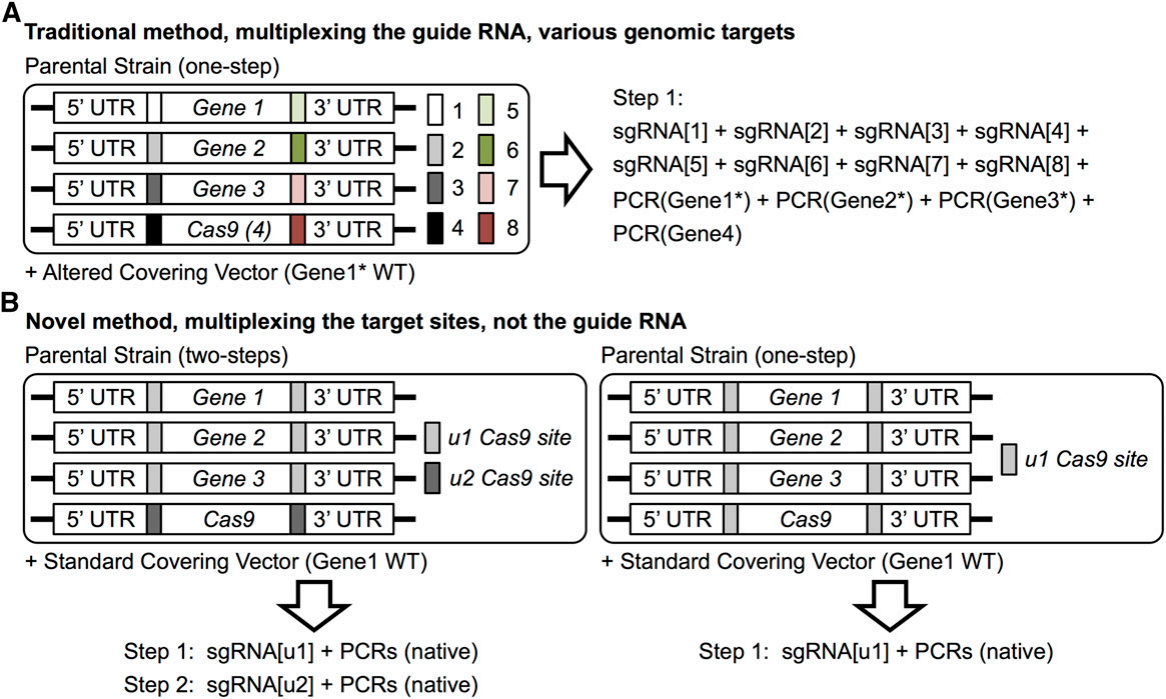
Comparison of Cas9-mediated genome editing by multiplexing sgRNAs *vs.* multiplexing loci with a unique target site. (A) Traditional targeting of Cas9 to multiple genomic loci (including one locus where Cas9 is integrated). Each of four loci is illustrated as requiring Cas9 action at two distinct sites. Hence, concurrent action of Cas9 at these four genes would require the selection of eight individual PAM-containing genomic sequences and the production of eight corresponding sgRNAs. In addition, it should be noted that, in this scenario, at least one target site lies within the coding sequence of each gene; therefore, PCR fragments used to replace Genes(1–3) would also require alterations of the coding sequence to avoid recutting by Cas9 (also see Figure S1). Finally, for manipulation of any essential genes (*e.g.*, Gene1), a counterselectable plasmid expressing a WT copy will also need to be altered to not include the genomic target site(s), again to avoid its Cas9-mediated cleavage (Figure S1). (B) The approach of multiplexing the target site(s) has a number of useful advantages. First, there is no need to restrict the target for Cas9 cleavage to sequences that exist within the genome of interest, which may be suboptimal (with regard to off-target effects) or may have a limited number or inopportune placements of available PAM sites. Second, the artificial target site chosen for insertion may be any stretch of 23 nucleotides (20 plus a 5′-NGG-3′ PAM) taken from any known species (or designed *de novo*), as long as it has no counterpart in the genome of interest. In fact, such a programmed target site sequence should greatly reduce or eliminate off-target effects, and also has the virtue that it can be inserted at a precise location (down to the base pair) to optimally facilitate recombination and precisely control the placement of the Cas9-mediated DSBs. The limiting step in this approach is, of course, introduction of these unique target site insertions into the parental genome at the desired locations. Once created, however, such an engineered parental strain can be used repeatedly to install various different alterations at one or many loci using only a single sgRNA, allowing for rapid construction of multiple strain variants. Moreover, in this approach, the Cas9 expression cassette can be retained, targeted for simultaneous excision in parallel with the manipulations of other loci (*right*), or eliminated at a later time, if the Cas9 expression cassette is flanked with a separate unique target site (*left*). Finally, because the sequence of the target sites flanking each locus are distinct from any of the elements of the targeted genes themselves, no modifications to the sequence of the PCR fragments used for gene editing (or of a covering plasmid carrying the corresponding WT gene) are required to make them immune to the further action of Cas9. DSB, double-strand break; PAM, protospacer adjacent motif; PCR, polymerase chain reaction; sgRNA, single-guide RNA; UTR, untranslated region; WT, wild-type.

The crux of our method is first programming the desired Cas9 cleavage sites at will by installing a unique sequence of the investigator’s own choosing, rather than relying on naturally-occurring genomic sequences (Figure 4). By flanking any number of selected genes with the same “alien” sequence, and providing PCR fragments homologous to the loci of interest, expression of just a single sgRNA initiates multiplex Cas9-mediated removal and scarless replacement of these targeted genes. Here, we achieved concurrent replacement of three ORFs on three different chromosomes, including one essential gene; however, the same approach can be used to excise or alter exons, introns, splice junctions, transcription factor-binding sites, locus control regions, etc. Our method also eliminates the need for codon alterations to the integrated allele(s) [or any WT covering plasmid(s)] to prevent recutting of the newly substituted DNA by Cas9 (Figure S1).

Other methods for modifying essential genes in yeast (Toh-e and Oguchi 2000; Cross and Pecani 2011; Horwitz *et al.*, 2015) have been described . In our view, our approach provides an alternative that is, in the long run, substantially less cumbersome and markedly more efficient. For example, our strategy does not depend on the fortuitous presence of a unique restriction endonuclease site, as required by the “integration replacement/disruption” method of Toh-e and Oguchi (2000) to “loop-in” a mutagenized plasmid copy of the gene of interest. Although the HO endonuclease-based method of Cross and Pecani (2011) does not require selection, it does require the construction of strain backgrounds with inducible HO expression and demands the exclusive use of the pRS400 series of *ARS*-less and *CEN*-less integration vectors. In the use of Cas9 for editing of essential genes described by Horwitz *et al.* (2015), the endogenous target sequence used for DSB formation needs to lie as close as possible to the desired nucleotide change to prevent inappropriate HR downstream of the mutation resulting in repair of the DSB without incorporation of the desired allele, as we already pointed out (Figure S1). Our approach circumvents all of the above issues, as well as increases the ease and efficiency by which essential genes may be manipulated.

Our methodology is complementary to “traditional” Cas9-mediated multiplex gene editing that requires the design and expression of multiple sgRNAs. Our approach expands how Cas9-based genome editing technology can be deployed and, hence, enhances its utility. Although our strategy first requires the initial installation of a unique target site(s) within the genome to be manipulated, there are several long-term benefits of constructing strains with programmed Cas9 target sites that we feel outweigh the traditional Cas9 approach (Figure 4 and Figure S1). Our method is especially useful (i) when repeated targeting of a locus, or groups of genes (*e.g.*, paralogs or entire genetic pathways) is needed, (ii) for manipulation of essential genes, (iii) where minimizing off-target effects is critical, and (iv) in cases were an “alien” target sequence is required/desired, such as in the design of gene drives. Given the rapid movement toward programmable toolkits in synthetic biology, we envision that it would be worth investing the effort to flank every gene in a genome of interest with such synthetic Cas9 target sites. In fact, traditional Cas9 editing could be used to do so.

Moreover, as we have demonstrated, Cas9 action at the artificially introduced sites can eliminate its own expression cassette without compromising its ability to mediate efficient gene editing elsewhere in the genome of the same cell. In addition, our method can be used to interrogate the effects of chromosome position and local chromatin structure on Cas9 action, because the same 23 bp sequence can be installed at any location in a genome. In this way, apparent differences between species with regard to the efficiency with which Cas9 can access and cleave at sites within heterochromatin (Yu *et al.* 2013; Wu *et al.* 2014; Knight *et al.* 2015; Feng *et al.* 2016) could be systematically explored. Finally, application of this approach should be extremely useful in generating strain libraries, constructing synthetic genomes, and introducing in a multiplex manner genomic changes to study multiple genes in a signaling pathway, the subunits of a multi-protein complex, paralogous gene sets, or any combination or collection of genes of interest.

## Supplementary Material

Supplemental Material
