# Corrigendum

**DOI:** 10.1534/g3.119.400096

**Published:** 2019-03-28

In the article by G. C. Finnigan and J. Thorner (*G3: Genes|Genomes|Genetics* 6 (7): 2147-2156) entitled “mCAL: A New Approach for Versatile Multiplex Action of Cas9 Using One sgRNA and Loci Flanked by a Programmed Target Sequence”, the authors wish to update the Acknowledgments with the following information.

G. C. F. and J. T. were inventors for a patent (Application No. PCT/US2017/028676; “Methods and Compositions for Genomic Editing”) filed on April 20, 2017 by the University of California, Berkeley. The authors declare no other conflict of interest.

