# Supplementary material for: mCAL: A New Approach for Versatile Multiplex Action of Cas9 Using One sgRNA and Loci Flanked by a Programmed Target Sequence
Source: G3 (Bethesda). 2016 May 13;6(7):2147–56. doi: 10.1534/g3.116.029801 (PMC4938667; doi:10.1534/g3.116.029801)
Supplement: Supplemental Material [file supp_6_7_2147__index.html]

mCAL: A New Approach for Versatile Multiplex Action of Cas9 Using One sgRNA and Loci Flanked by a Programmed Target Sequence — Supplemental Material 

# mCAL: A New Approach for Versatile Multiplex Action of Cas9 Using One sgRNA and Loci Flanked by a Programmed Target Sequence

## Supplemental Material for Finnigan and Thorner, 2016

**Files in this Data Supplement:**

- Figure S1 - Limitations of "classical" Cas9-mediated manipulation of essential genes. (.pdf, 144 KB)
- Figure S2 - Expression of Cas9-eGFP and the benign effect of the programmable flanking site system *in vivo*. (.pdf, 330 KB)
- Figure S3 - Concurrent integration at either of two (out of three) genomic loci with concomittant removal of Cas9 using the single sgRNAu1 system and only 30 bps of flanking homology. (.pdf, 547 KB)
- Figure S4 - Possible outcomes of incomplete Cas9-targeting of u1 sites at the *CDC11* locus *in vivo*. (.pdf, 132 KB)
- Figure S5 - Analysis of very rare surviving isolates from controls where one or more PCR fragments for integration were not provided. (.pdf, 554 KB)
- Table S1 - Yeast strains used in this study. (.pdf, 96 KB)
- Table S2 - Plasmids used in this study. (.pdf, 87 KB)
- Table S3 - Transformation efficiency for controls lacking expression of either Cas9 or sgRNA. (.pdf, 86 KB)
- Table S4 - Growth results for colonies from controls lacking at least one PCR product. (.pdf, 84 KB)
- Table S5 - Oligonucleotides used in this study. (.pdf, 84 KB)
- File S1 - Supplemental References (.pdf, 87 KB)
