# Supplementary material for: mCAL: A New Approach for Versatile Multiplex Action of Cas9 Using One sgRNA and Loci Flanked by a Programmed Target Sequence
Source: G3 (Bethesda). 2016 May 13;6(7):2147–56. doi: 10.1534/g3.116.029801 (PMC4938667; doi:10.1534/g3.116.029801)
Supplement: Supplemental Material [file supp_g3.116.029801_FigureS1.pdf]

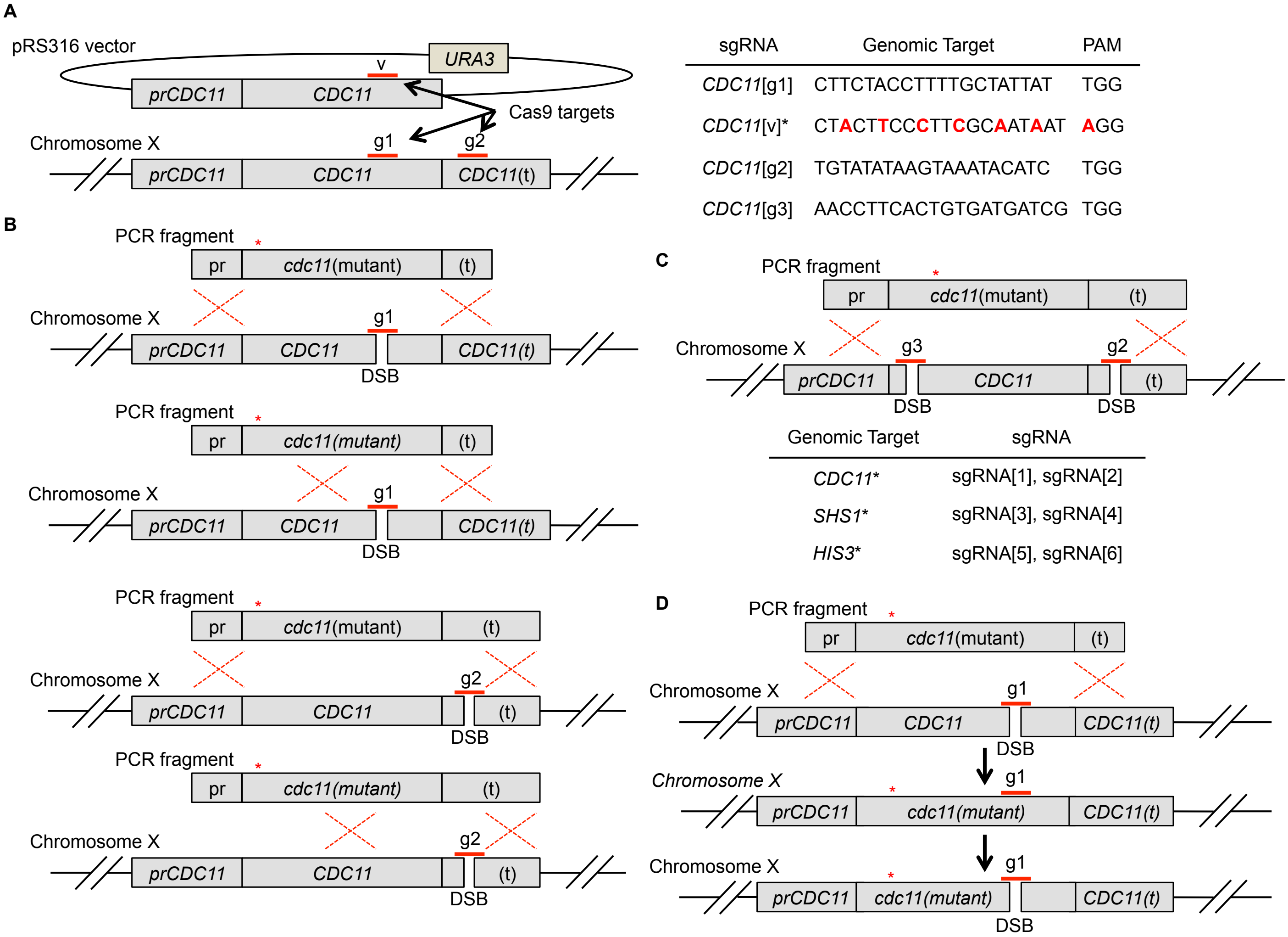

FIGURE S1. Limitations of “classical” Cas9-mediated manipulation of essential genes. (A) Targeting an essential gene *in vivo* requires the presence of a WT-expressing “covering” plasmid that can be counter-selected (harboring *URA3*) by growth on medium containing 5-FOA (BOEKE *et al.* 1984). Targeting the *CDC11* gene at two genomic positions, g1 (within the coding region) and g2 (within the flanking 3’ UTR) requires that both 23-nucleotide sequences are not present within the WT copy of the covering plasmid expressing *CDC11*. Example Cas9 target site sequences are shown based on the proposed collection of target sites within the yeast genome (DICARLO *et al.* 2013). If the g1 target sequence within *CDC11* were used, the corresponding sequence of the covering vector would have to be mutated to prevent inappropriate targeting by Cas9 to the vector sequence. Synonymous changes could be made (red text) to the covering vector, so as to not alter the amino acid sequence (target sequence v) with a priority to the nucleotides within the PAM motif and the 12 bps immediately upstream sequence, as these are the most significant for Cas9 target site “seeding” (JINEK *et al.* 2012; JIANG *et al.* 2013). Since the covering vector does not contain the native 3’-UTR of *CDC11*, no additional changes need to be made to allow for targeting at g2 position within the terminator. However, it should be noted that 3’-UTR sequences may pose difficult in finding suitable Cas9 target sites due to the AT-rich stretches, and the possibility for a TTTTTT sequence which can terminate RNA polymerase III transcripts (BRAGLIA *et al.* 2005; WANG AND WANG 2008) and lower GC content within the target sequence. (B) If a single genomic target is used, g1 or g2, to create a double-stranded break (DSB) within the *CDC11* locus, there are several scenarios that would allow for inappropriate crossover when integrating a mutant allele (red asterisk) in place of the WT endogenous copy. When using g1, which is downstream of the proposed mutant allele, the crossover event could occur either upstream of the mutation within the promoter

sequence (first panel) or downstream of the mutation within the coding sequence (second panel). The second event would repair the DSB, but fail to integrate the mutant allele. Similarly, use of g2 within the terminator sequence would also have the same issue with the possibility of the crossover event occurring downstream of the desired mutation. Thus, in order to optimize the integration efficiency of the mutant allele, one should (i) increase the 5'-homology (promoter) sequence and/or (ii) choose a Cas9 target site as close to the mutation as possible to prevent repair of the WT gene copy, as also suggested by others (HORWITZ *et al.* 2015). However, if the mutation is near the initiator Met within the coding sequence, or the coding sequence of the gene is long, then choosing a g2 terminator target (which is not present within the covering vector) encounters the same efficiency issues. Therefore, programming Cas9 to target a specific position close to the desired mutation g1 would increase integration efficiency, but would *also* require changing the same target site within the covering vector sequence v. Furthermore, if multiple mutant alleles spanning the entire length of the gene were to be integrated, this strategy would either require multiple Cas9 genomic targets, or varying efficiencies based on the position of the desired mutation(s). Additionally, for essential genes, the presence of the covering vector creates an additional step (to mutate the WT gene sequence) to prevent Cas9-dependent targeting of this protective vector. (C) One possible solution is to target multiple positions within the locus of interest and create two DSBs, one of which is upstream of the mutation of interest within the coding sequence g3. In this way, recombination can *only* occur upstream and downstream of the two DSBs, and would efficiently introduce the desired allele with virtually no inappropriate crossover events (assuming efficient targeting by Cas9). However, since g3 is still within the coding sequence, the v position within the covering vector would require alteration and rather than creating a single sgRNA construct, two constructs sgRNA[g2] and sgRNA[g3] would be required to manipulate this single locus. The amount of cloning required would be further increased if this strategy were extended to additional loci; targeting of three genes would require multiplexing 6 guide RNA cassettes *and* the covering vector sequences (see Fig. S1C, *lower*). (D) Finally, integration of an essential gene allele using a traditional Cas9 approach requires yet another cloning step. If any genomic target is chosen within the coding sequence, g1 or g3 for instance, then the coding sequence of the integrated allele itself at these same position(s) must *also* be altered so as to prevent Cas9-mediated targeting of the final integrated allele. Therefore, at minimum, a single guide g1 strategy requires (i) alteration of the covering vector, (ii) alteration of the mutant allele itself, and allows for (iii) inappropriate crossover between the mutant allele and g1 target site, and (iv), would likely have to be altered for various alleles or constructs spanning the length of the target gene. A two-guide strategy, targeting g2 and g3, which removes the possibility of inappropriate crossover, requires the same cloning alterations including the vector, the integrated allele itself, *and* design and construction of two sgRNA cassettes. These drawbacks make the traditional Cas9 strategy laborious, suboptimal, and require the separate construction of many components aside from Cas9 and the sgRNAs themselves.
