# Supplementary material for: mCAL: A New Approach for Versatile Multiplex Action of Cas9 Using One sgRNA and Loci Flanked by a Programmed Target Sequence
Source: G3 (Bethesda). 2016 May 13;6(7):2147–56. doi: 10.1534/g3.116.029801 (PMC4938667; doi:10.1534/g3.116.029801)
Supplement: Supplemental Material [file supp_g3.116.029801_FigureS2.pdf]

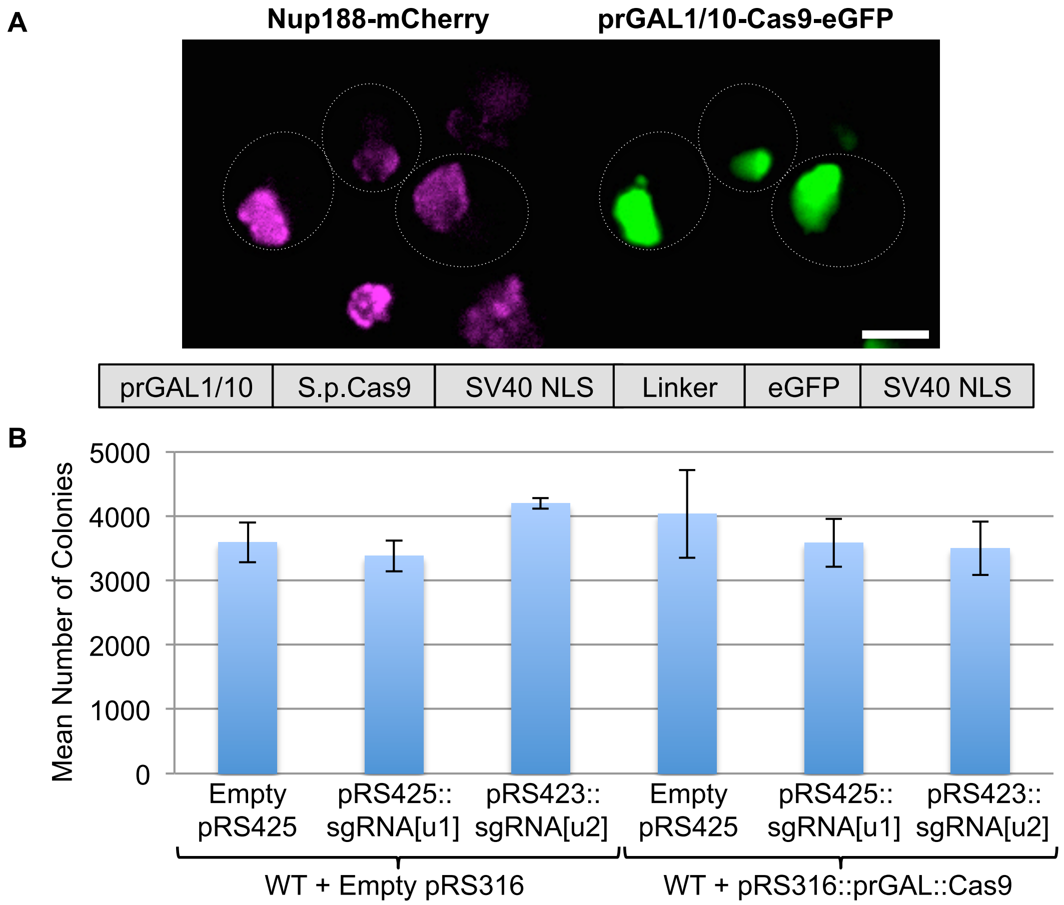

FIGURE S2. Expression of Cas9-eGFP and the benign effect of the programmable flanking site system *in vivo*. (A) Yeast (GFY-1517) expressing Nup188-mCherry (marker for the nuclear envelope) were transformed with a plasmid (pGF-IVL977) expressing under control of the *GAL1/10* promoter S.p.Cas9 containing an SV40 NLS sequence (SRADPKKKRKV), a flexible SGGGSG linker, eGFP, and a second SV40 NLS, as indicated, were grown overnight in S+Raf/Suc-Leu, back-diluted in YP+Gal, grown at 30˚C for 3 h, and imaged by fluorescence microscopy. Representative cells are shown. White dotted lines, cell periphery; scale bar, 2 μM. (B) Expression of Cas9 alone, or either sgRNA[u1] or sgRNA[u2] alone, or expression of Cas9 with either sgRNA[u1] or sgRNA[u2], did not result in any marked differences in transformation efficiency or viability of the resulting yeast colonies. WT cells (BY4741) were first transformed with either an empty pRS316 vector or a pRS316::prGAL1/10::Cas9 vector (pGF-V789). Next, the yeast were grown overnight in S+Raf/Suc-Ura medium to saturation, back-diluted into YP+Gal, grown at 30˚C for 5 h, and then transformed with equal amounts (1-2 μg) of empty pRS425, pRS425::sgRNA[u1], or pRS423::sgRNA[u2] vectors. After recovery overnight in YP+Gal, the cells were plated onto SD-Ura-Leu (for pRS425 vectors) or SD-Ura-His (for pRS423 vectors). Experiments were performed in triplicate; colonies were counted and the average colony number was plotted for each genotype (error bars, SEM). There was no statistically significant difference between WT yeast (*first strain;* unpaired t-test p-value = 0.59) and yeast over-expressing Cas9 (*fourth strain*). Similarly, there was no statistically significant difference between expression of sgRNA[u1] alone (*second strain*) compared to co-expression with Cas9 (*fifth strain*; p-value = 0.67). Similarly, there was no statistically significant difference with or without Cas9 for sgRNA[u2] (*third and sixth strains*; p-value = 0.18). These data suggest (i) that Cas9 expression *per se* is not toxic in yeast, as has been previously reported (DICARLO *et al.* 2013) and (ii) that neither sgRNA[u1] or sgRNA[u2] are toxic to yeast (*i.e.*, they do not mediate formation of any lethal DSB at off-target locations within the yeast genome).
