# Supplementary material for: mCAL: A New Approach for Versatile Multiplex Action of Cas9 Using One sgRNA and Loci Flanked by a Programmed Target Sequence
Source: G3 (Bethesda). 2016 May 13;6(7):2147–56. doi: 10.1534/g3.116.029801 (PMC4938667; doi:10.1534/g3.116.029801)
Supplement: Supplemental Material [file supp_g3.116.029801_FigureS3.pdf]

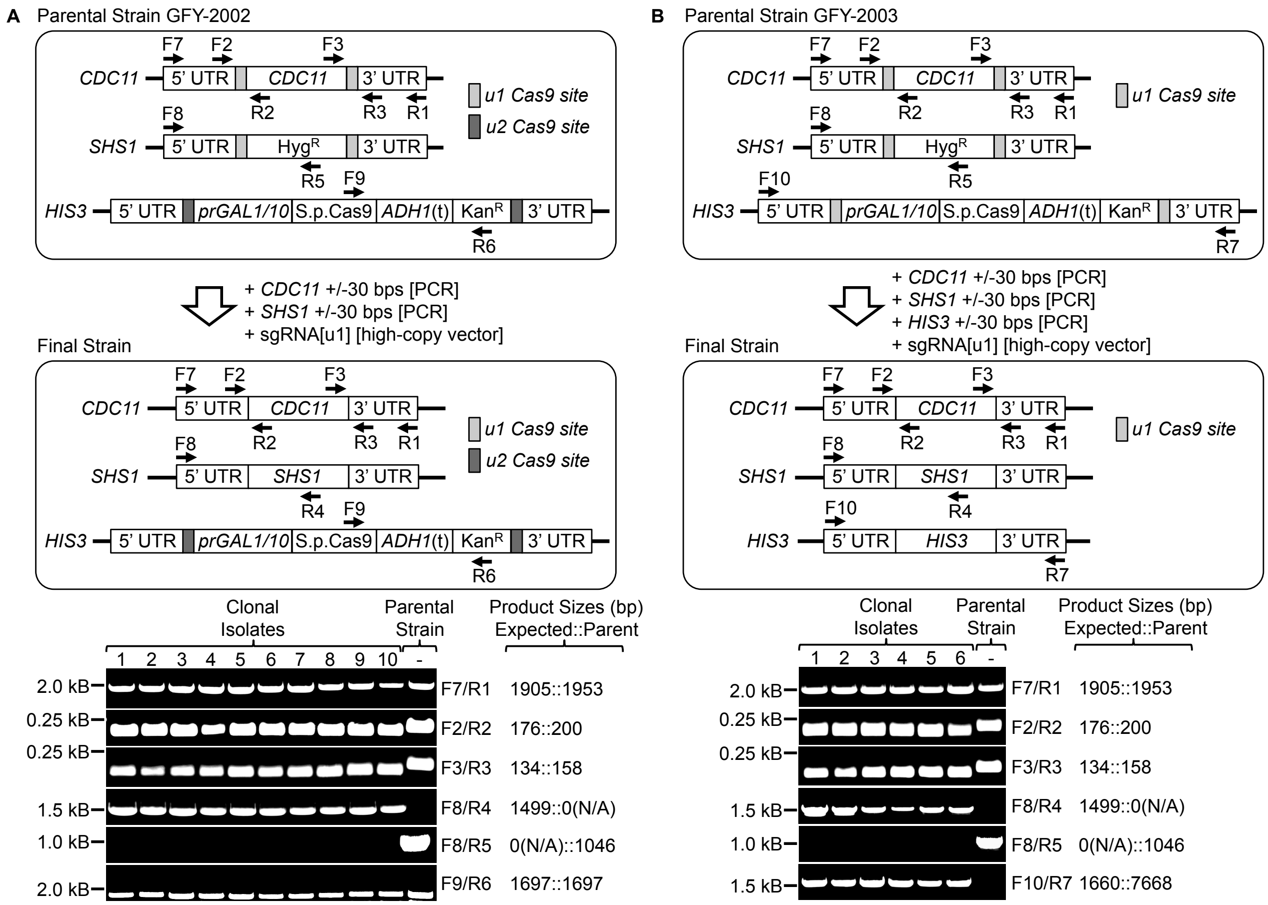

FIGURE S3. Concurrent integration at either of two (out of three) genomic loci with concomittant removal of Cas9 using the single sgRNA[u1] system and only 30 bps of flanking homology. (A) Strain GFY-2002 was induced for Cas9 expression and transformed with the sgRNA[u1] expression vector and PCR products for the *CDC11* and *SHS1* loci, as in Fig. 2. Each PCR fragment was flanked by 30 bps of homology to the genomic target. Chromosomal DNA was purified (AMBERG *et al.* 2006) from ten representative clonal isolates (chosen on the basis of their phenotype, assessed as in Table S4), and diagnostic PCRs were performed and the resulting DNA products analyzed by agarose gel electrophoresis. For *CDC11* (*top three gels*), the entire locus was amplified (oligos F7/R1) as well as small fragments flanking either the upstream u1 site (F2/R2) or the downstream u1 site (F3/R3) to determine if the 23-base pair Cas9 target site was still present or not. For *SHS1* (*fourth and fifth gels*), a PCR (F8/R4) was performed with a unique primer internal to the *SHS1* coding region that was not present within the parental strain, as well as a PCR containing a unique primer to the Hyg^R cassette that was present in the parental strain, but not in any putative integrant that repaired the *SHS1* gene. Finally, the *HIS3* locus was tested with a unique primer internal to the Cas9 gene and to the *HIS3* 3'-UTR (F9/R6) to illustrate this genomic site was unperturbed (it was programmed with flanking u2 sequences). For optimal separation on the second and third DNA gels, a 2% agarose mixture was used compared to the standard 1% concentration. DNA size markers, kB (kilobases). Finally, the expected fragment size for each diagnostic PCR is shown for each reaction. (B) An analysis identical to that in (A) was performed using parental strain GFY-2003 (which has u1 sites flanking the Cas9 cassette at the *HIS3* locus). Six independent clonal isolates were obtained and tested as in (A), except that one PCR fragment was included that contained the WT *HIS3* gene (also with 30 bps of flanking UTR) to eject and replace the endogenous Cas9 cassette. To monitor substitution of the Cas9 cassette with the repaired *HIS3* gene, PCR for the entire *HIS3* locus was performed (F10/R7). At least one of the two primers used for each diagnostic PCR corresponds to sequences that lie outside the region contained within the PCR fragments used for transformation.
