# Supplementary material for: mCAL: A New Approach for Versatile Multiplex Action of Cas9 Using One sgRNA and Loci Flanked by a Programmed Target Sequence
Source: G3 (Bethesda). 2016 May 13;6(7):2147–56. doi: 10.1534/g3.116.029801 (PMC4938667; doi:10.1534/g3.116.029801)
Supplement: Supplemental Material [file supp_g3.116.029801_FigureS4.pdf]

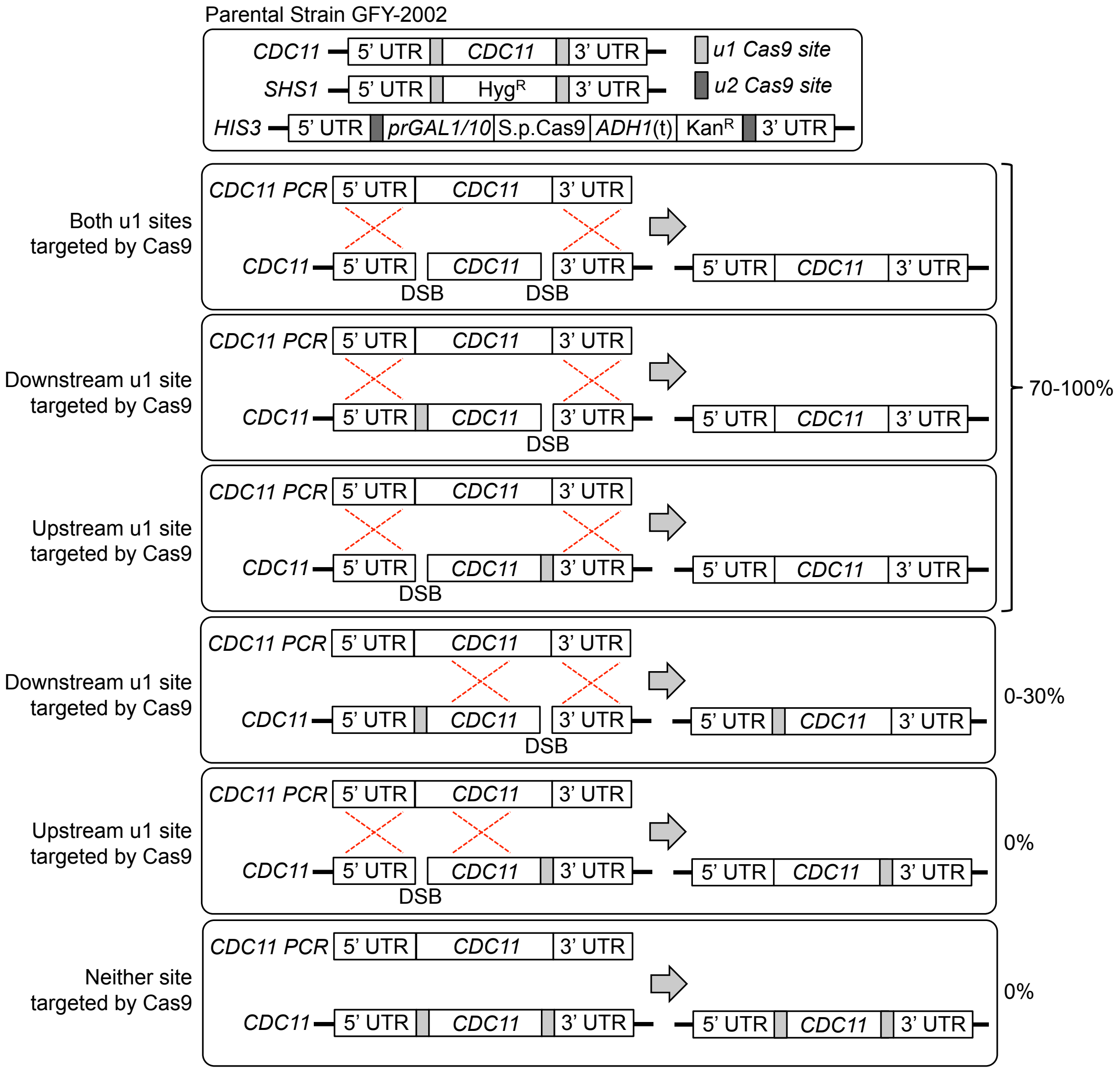

FIGURE S4. Possible outcomes of incomplete Cas9-targeting of u1 sites at the *CDC11* locus *in vivo.* Although the majority of the isolates tested displayed proper replacement of the u1-flanked *CDC11* locus with the WT *CDC11* gene carried on the PCR fragment, a small percentage of the isolates tested showed replacement of the downstream u1 site, but not the upstream u1 site (see Fig. 3). However, the *CDC11* locus replacement differed from that of *SHS1*, or *HIS3* (for parental strain GFY-2003 only), in that the genomic target had a sequence identical to that provided on the PCR fragment. For *SHS1*, the WT gene replaced the Hyg^R deletion, and for *HIS3*, the WT gene replaced the Cas9 cassette. If Cas9 targeted either or both of the u1 sites at the *CDC11* locus and the crossover events occurred outside of the coding sequence (*top three panels*), then the full WT *CDC11* gene would replace the parental u1-flanked cassette and neither Cas9 target site would remain, as we observed for 70-100% of the isolates screened in four independent experiments. If, by contrast, Cas9 failed to create any DSB at the *CDC11* locus, then both u1 sites would remain; reassuringly, none of the isolates tested displayed this pattern. If Cas9 only targeted one u1 genomic location at the *CDC11* locus, *and* the crossover occurred within the *CDC11* coding region, then the DSB would be repaired by PCR product provided, but one u1 site would remain intact. Out of 36 total isolates tested, 100% had the downstream u1 site removed, whereas just four retained the upstream site u1 site, which may reflect some difference in chromatin structure or occupancy by RNA polymerase or something else that affects their accessibility to Cas9.
