# Supplementary material for: mCAL: A New Approach for Versatile Multiplex Action of Cas9 Using One sgRNA and Loci Flanked by a Programmed Target Sequence
Source: G3 (Bethesda). 2016 May 13;6(7):2147–56. doi: 10.1534/g3.116.029801 (PMC4938667; doi:10.1534/g3.116.029801)
Supplement: Supplemental Material [file supp_g3.116.029801_FigureS5.pdf]

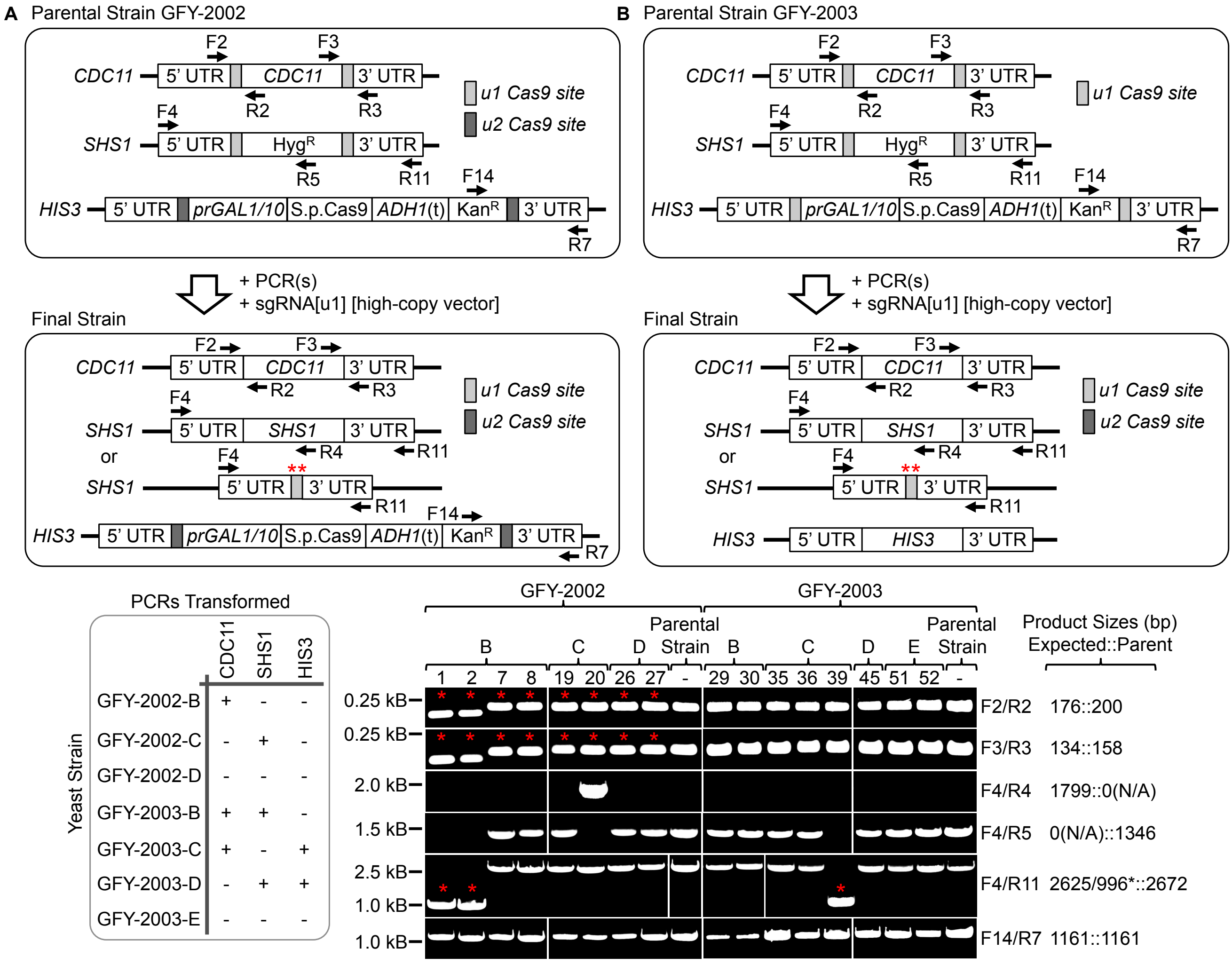


**FIGURE S5**. Analysis of very rare surviving isolates from controls where one or more PCR fragments for integration were not provided. (A and B) Clonal isolates from multiple experimental trials (Fig. 2B) were tested by both growth (all tested isolates; Table S4) and diagnostic PCR (1 to 4 isolates chosen from each growth category). A summary of the yeast strains used and the PCR fragment(s) added in each transformation are summarized (*lower left*). Primers flanking the *CDC11* u1 Cas9 sites (F2/R2 and F3/R3) were used for PCR to determine whether the WT *CDC11* allele has been integrated (*top two gels*). For the *SHS1* locus, PCR with a unique primer to the *SHS1* coding sequence (R4, *third gel*) or to the Hyg$^R$ cassette (R5, *fourth gel*) were used. Because isolates 1, 2, and 39 appeared to have neither the WT *SHS1* gene nor the deletion cassette, primers flanking the *SHS1* locus (F4/R11) were used for amplification (*fifth gel*). If the full *SHS1* locus has been deleted, the diagnostic PCR product is expected to be 996 bps (*product size, black asterisk*). Finally, the *HIS3* locus was interrogated by PCR (F14/R7, *sixth gel*). Selected PCR products (*red asterisk*) were purified and sequenced. For isolates 1, 2, and 39, DNA sequencing revealed that the deletion present removed the *SHS1* locus, but left behind a single u1 site (*double red asterisk*), suggesting that after excision of the *SHS1* locus, the break was repaired by recombination between the two cut u1 sites. The diagnostic PCR products were analyzed on 2% agarose gels (*first and second gels*) or 1% (*gels three through six*); DNA size markers, kB (kilobases). For products resolved on different gels, the gel fragments are shown as separate images. All images were scaled identically and are marked with both DNA ladders and the predicted PCR product sizes from the parental strains GFY-2002 or GFY-2003, as indicated) using the same primer set.
