# Supplementary material for: mCAL: A New Approach for Versatile Multiplex Action of Cas9 Using One sgRNA and Loci Flanked by a Programmed Target Sequence
Source: G3 (Bethesda). 2016 May 13;6(7):2147–56. doi: 10.1534/g3.116.029801 (PMC4938667; doi:10.1534/g3.116.029801)
Supplement: Supplemental Material [file supp_g3.116.029801_TableS1.pdf]

Table S1. Yeast strains used in this study.

Strain	Genotype	Reference
BY4741	*MAT**a** leu2Δ ura3Δ met15Δ his3Δ*	(Brachmann *et al.* 1998)
GFY-1517[1]	*BY4741; NUP188::mCherry::ADH1(t)::S.p.HIS5*	This study
GFY-2002[2]	*BY4741; cdc11Δ::u1::CDC11::u1; shs1Δ::u1::Hyg^R::u1; his3Δ::u2::prGAL1/10^3::S.p.Cas9::NLS::ADH(t)::Kan^R::u2 + pJT1520*	This study
GFY-2003[4]	*BY4741; cdc11Δ::u1::CDC11::u1; shs1Δ::u1::Hyg^R::u1; his3Δ::u1::prGAL1/10::S.p.Cas9::ADH(t)::Kan^R::u1 + pJT1520*	This study

[1]Wild-type (WT) yeast was transformed with an amplified PCR product (from plasmid pJT2868) to tag the endogenous copy of *NUP188*, a nuclear envelope protein (Aitchison *et al.* 1995; Fabre and Hurt 1997), with mCherry. The *S.p.HIS5* gene is from fission yeast *S. pombe* (and functions in place of *S. cerevisiae HIS3*).

[2]To construct GFY-2002, yeast strain GFY-153 (*cdc11Δ::Kan^R* + *pRS316::CDC11*) was transformed with the amplified *CDC11* product (from pGF-IVL972) containing both flanking u1 Cas9 sites in-frame with the ORF as well as 330 bps of 5'- and 3'-UTR and plated on synthetic complete medium containing 5-FOA (to select for the integration of the u1::CDC11::u1 copy and loss of the WT Cdc11-expressing *URA3*-based plasmid). The *CDC11*-expressing *URA3* plasmid (pJT1520) was subsequently transformed back into an isolate carrying the desired u1::*CDC11*::u1 integrant. Second, *SHS1* was deleted using a modified Hyg^R deletion cassette (Goldstein and McCusker 1999) containing flanking u1 sites (23 bps) upstream and downstream of the MX sequence (from pGF-IVL1026) with 500 bps of *SHS1* UTR. Third, the *HIS3 locus* (*his3Δ0*) was repaired by amplifying a WT copy of *S.c.HIS3* with 500 bps of flanking UTR by PCR (template was genomic DNA from THS4213) and selected on SD-His medium. Fourth, the expression cassette for S.p.Cas9 (Cas9 from *S. pyogenes*; human codon bias; amplified from Addgene plasmid #43804) was integrated at the *HIS3* locus by PCR amplifying the following fragment in two, roughly equal-sized pieces (of approximately 5 kb each) that overlapped within the Cas9 gene, *prHIS3::u2::prGAL1/10::S.p.Cas9::ADH(t)::Kan^R::u2::HIS3-3'UTR* (from pGF-IVL975), and selected on rich medium containing G418. The Cas9 gene has the SV40 NLS signal (Kalderon *et al.* 1984) appended at its C-terminus. Following each chromosomal integration event, genomic DNA was purified, amplified by PCR, and confirmed by Sanger sequencing for all three manipulated loci (*CDC11, SHS1,* and *HIS3*) including their 5'-

and 3'-UTRs and the presence of each u1 and/or u2 Cas9 target sites.

[3]814 base pairs of the *prGAL1/10* promoter were used upstream of the initiator Met to overexpress Cas9.

[4]GFY-2003 was created similarly to GFY-2002, but used a Cas9-expressing cassette containing the flanking u1 sites rather than u2 (amplified from pGF-IVL1027), but is otherwise isogenic.
