# Supplementary material for: mCAL: A New Approach for Versatile Multiplex Action of Cas9 Using One sgRNA and Loci Flanked by a Programmed Target Sequence
Source: G3 (Bethesda). 2016 May 13;6(7):2147–56. doi: 10.1534/g3.116.029801 (PMC4938667; doi:10.1534/g3.116.029801)
Supplement: Supplemental Material [file supp_g3.116.029801_TableS2.pdf]

Table S2. Plasmids used in this study.

Plasmid	Description	Reference
pRS425	2µ; *LEU2 AMP*	(CHRISTIANSON *et al.* 1992)
pRS423	2µ; *HIS3 AMP*	(CHRISTIANSON *et al.* 1992)
pRS316	CEN; *URA3 AMP*	(SIKORSKI AND HIETER 1989)
pSB1/JT1520	CEN; *URA3 AMP*; *prCDC11::CDC11*[1]	(VERSELE *et al.* 2004)
pGF-V796[2]	pRS425; sgRNA[u1]	This study
pGF-V798[3]	pRS423; sgRNA[u2]	This study
pGF-IVL977[4]	*prHIS3::u2::prGAL1/10::S.p.Cas9::NLS::Linker::eGFP::NLS::ADH(t)::Kan^R::u2::HIS3-3'UTR*	This study
pGF-V789[5]	pRS316; *prGAL1/10::S.p.Cas9::NLS::CDC10-3'UTR*	This study

[1]There is no *CDC11* 3'-UTR present within this vector.

[2]The sgRNA[u1]-expressing cassette is under control of the snoRNA *SNR52* promoter and *SUP4* terminator sequences (DICARLO *et al.* 2013). The u1 target sequence is CGGTGGACTTCGGCTACGTA. The entire sgRNA-expressing cassette was synthesized (GenScript, Piscataway, NJ) with flanking *BamHI* and *XhoI* restriction sites in the vector pUC57 (GenScript Cat. No. SD1176; GenBank Y14837.1; A. Markauskas and G. Dreguniene, unpublished) and subcloned to pRS425 followed by sequence verification.

[3]The sgRNA[u2]-expressing cassette was mutated from the u1 sequence within pUC57 through successive rounds of PCR mutagenesis (ZHENG *et al.* 2004) to generate a u2 target sequence of GCTGTTCGTGTGCGCGTCCT followed by a final subcloning into vector pRS423.

[4]Plasmid pGF-IVL977 was constructed by first creating a parent vector by *in vivo* ligation and homologous recombination in yeast (FINNIGAN AND THORNER 2015) of the genotype: *prHIS3::u2::prGAL1/10::NotI restriction site::ADH1(t)::KanR:u2::HIS3-3'UTR* (pGF-IVL974). Next, a second round of *in vivo* ligation in yeast was used to insert the *S.p.Cas9::NLS::Linker::eGFP::NLS* sequence. The flexible linker has the sequence SGGGSG and the SV40 NLS sequence (KALDERON *et al.* 1984) is SRADPKKKRKV and is found after both the Cas9 and eGFP sequences. Under identical induction and growth conditions, a similar construct (pGF-IVL976) with only a single SV40 NLS present between Cas9 and a C-terminal eGFP tag did not yield as strong of a fluorescent signal within yeast cell nuclei compared to the

pGF-IVL977 construct containing two SV40 nuclear localization signals.

[5]This vector was constructed first by *in vivo* ligation in pRS315 to fuse the *prGAL1/10* promoter, Cas9 gene, *CDC10* 3'-UTR terminator (465 bps), and the Kan^R cassette with a unique *SpeI* site present between the terminator and drug cassette. A unique *NotI* site (upstream of the *prGAL1/10* sequenece) was used to subclone the *prGAL1/10::Cas9::NLS::CDC10(t)* sequence to the same sites in pRS316 to yield pGF-V789.
