# Supplementary material for: mCAL: A New Approach for Versatile Multiplex Action of Cas9 Using One sgRNA and Loci Flanked by a Programmed Target Sequence
Source: G3 (Bethesda). 2016 May 13;6(7):2147–56. doi: 10.1534/g3.116.029801 (PMC4938667; doi:10.1534/g3.116.029801)
Supplement: Supplemental Material [file supp_g3.116.029801_TableS3.pdf]

Table S3. Transformation efficiency for controls lacking expression of either Cas9 or sgRNA.

Yeast Strain	Growth[1]	Plasmid transformed	Colonies[2]	Trials
GFY-2002	Galactose	Empty pRS425	1614[2] +/- 427[3]	n=7
GFY-2002	Dextrose	pRS425::sgRNA[u1]	6025 +/- 4260	n=2
GFY-2003	Galactose	Empty pRS425	1310 +/- 186	n=7
GFY-2003	Dextrose	pRS425::sgRNA[u1]	5996 +/- 4239	n=2

[1]Yeast cultures were grown overnight in S+Raf/Suc-URA, back diluted (to an $A_{60o\ nm}$ of roughly 0.3) in YP+Gal and incubated for 5 h at 30°C. The cells were transformed with equal amounts (1-2 µg) of empty pRS425 vector or pRS425::sgRNA[u1] using a modified lithium acetate protocol (ECKERT-BOULET *et al.* 2012) and recovered overnight in YP+Gal medium prior to plating onto SD-Ura-Leu plates. Colonies from each experimental trial were counted after a 3-day incubation at 30°C. Resulting isolates were grown in either galactose-containing medium to induce expression of Cas9, or in dextrose-containing medium to inhibit Cas9 expression.

[2]Colony number was estimated by plating several dilutions (1:10, 1:20, 1:100, etc.) to selective plates and the average total colony count was reported.

[3]Error is SEM.

Quantification of gene replacement from Figure 2B:

For conversion of *shs1∆::Hyg*^{R} *to* WT *SHS1*, loss of hygromycin resistance was scored (for both GFY-2002 and GFY-2003); and, for conversion of the *his3∆::*Cas9::Kan^R cassette to WT *HIS3*, loss of G418 resistance and gain of ability to grow on SD-His medium were also scored (for GFY-2003 only). Because the u1-flanked chromosomal *CDC11* locus carried no markers, its conversion could not be scored by such a phenotypic analysis.

GFY-2002-A (500 bps flanking): 5 independent trials, 334 total colonies tested.

GFY-2002-A (30 bps flanking): 3 independent trials, 213 total colonies tested.

GFY-2003-A (500 bps flanking): 5 independent trials, 364 total colonies tested.

GFY-2003-A (30 bps flanking): 3 independent trials, 30 total colonies tested.

Quantification of gene replacement from Figure 2D:

For conversion of the *his3∆::*Cas9::Kan^R cassette to WT *HIS3* from a confirmed (repaired) isolate from GFY-2002-A (WT *CDC11* and WT *SHS1*) (Fig. 2B), colonies were tested for the ability to grown on SD-His medium and were also scored for G418 resistance.

GFY-2002-A (*CDC11 SHS1*) (pRS423: sgRNA[u2]): 3 independent trials and 196 independent colonies tested.

GFY-2002-A (*CDC11 SHS1*) (empty pRS423): 3 independent trials and 200 independent colonies tested.
