# Supplementary material for: mCAL: A New Approach for Versatile Multiplex Action of Cas9 Using One sgRNA and Loci Flanked by a Programmed Target Sequence
Source: G3 (Bethesda). 2016 May 13;6(7):2147–56. doi: 10.1534/g3.116.029801 (PMC4938667; doi:10.1534/g3.116.029801)
Supplement: Supplemental Material [file supp_g3.116.029801_TableS4.pdf]

Table S4. Growth results for colonies from controls lacking at least one PCR product.

Parental Strain	PCR fragment(s)	SD-Ura	SD-His	YPD +G418	YPD +Hyg	Total Colonies
2002-B[1]	*CDC11*	+	-	+	-	7
		+	-	+	+	9
2002-C	*SHS1*	+	-	+	-	2
		+	-	+	+	7
2002-D	-	+	-	+	-	0
		+	-	+	+	3
2003-B[3]	*CDC11 &*	+	-	+	-	0
	SHS1	+	-	+	+	6
2003-C	*CDC11 &*	+	-	+	-	2
	HIS3	+	-	+	+	7
2003-D	*SHS1 &*	+	-	+	-	0
	HIS3	+	-	+	+	6
2003-E	-	+	-	+	-	0
		+	-	+	+	6

Clonal isolates were pooled from multiple experimental trials, in which either 500 bps of flanking homology (Fig. 2) or 30 bps of flanking homology (Fig. S3) were used, and replica-plated onto various growth conditions (SD-Ura, SD-His, YPD+G418, and YPD+Hygromycin). A "+" score indicates growth/resistance whereas "-" designates no growth/sensitivity. For the initial genotypes indicated [strain and PCR fragment(s)], two growth patterns were observed and the number of colonies displaying each pattern were totaled (*far right column*). Yeast were also tested on SD-Leu medium for the presence or absence of the high-copy pRS425::sgRNA[u1] vector. After only two rounds of selection on SD-Ura (no selective pressure for the *LEU2* marker), 40/55 strains had lost the sgRNA-expressing plasmid. Representative isolates were taken from each category for further analysis by diagnostic PCR.

[1]B, C, D or E designation is from the experiment in Fig. 2, in which no PCR fragments, one PCR fragment, or two PCR fragments were introduced by transformation, as indicated.

[3]For simplicity, only combinations of two PCR products (omitting a third) were tested for strain GFY-2003, rather than all possible combinations.
