# Supplementary material for: mCAL: A New Approach for Versatile Multiplex Action of Cas9 Using One sgRNA and Loci Flanked by a Programmed Target Sequence
Source: G3 (Bethesda). 2016 May 13;6(7):2147–56. doi: 10.1534/g3.116.029801 (PMC4938667; doi:10.1534/g3.116.029801)
Supplement: Supplemental Material [file supp_g3.116.029801_TableS5.pdf]

Table S5. Oligonucleotides used in this study.

Oligonucleotide	Description	Sequence (5' to 3')
F1	*CDC11* UTR[1] +406 F	CATCTACAAAAGCAGGTTATAGCTCCG TTAAAC
F7	*CDC11* UTR +330 F	CACAACATGGAACATAACATTTAAACAT CGTTCTCAATC
R1	*CDC11* UTR -327 R	GCTAAGTGATGTTCTGGTCTTTCCAAAA TTCTC
F2	*CDC11* UTR +101 F	GATCGAAAAGTAAAATAGGTAGACACC ACGTATTG
R2	*CDC11* Int[2] +75 R1	CACAGTGAAGGTTATACCTCTTTTCAAA TGCTTTC
F3	*CDC11* Int +1233 F	GAAGCCAGGTTGGAAAAAGAGGCGAAA ATC
R3	*CDC11* UTR -89 R	TAATATACCAGATGTATTTACTTATATAC AACACTCACTC
F4	*SHS1* UTR +800 F	CATTACTAGAGTTTGCTGGTGTTGCAG ATCG
F8	*SHS1* UTR +500 F	GTTCCTCCGGTTTAAACTGATCCATAGT GATAG
R4	*SHS1* Int +999 R	ACGGTAATTCTCGTAAAGCAAATTTTGC GTGG
R5	Int Hyg R	CTCCTACATCGAAGCTGAAAGCACGAG
F5	Cas9 Int +2903 F	CTGAAGTCTAAGCTGGTCTCAGATTTCA GAAA
F9	Cas9 Int +3477 F	GTGGAGAAAGGGAAGTCTAAAAAACTC AAAAGC
R6	Int Kan R	GAACACTGCCAGCGCATCAACAATATT TTC
F6	*HIS3* UTR +992 F	GGGTCAGTTATTTCATCCAGATATAACC CG
F10	*HIS3* UTR +500 F	TTCTCGACGTGGGCCTTTTTCTTGCCAT AT

R7	*HIS3* UTR -497 R	GTAACCACCACGACGGTTGTTGCTAAAGAA
F11	*CDC11* UTR +30 F	CACAAGGCCTAAAGTTGCTAACCACCAGCCATGTCCGGAATAATTGACGCATCTTCTGCA
R8	*CDC11* UTR -30 R	AAGAAATAAGTGAGGAAGCCAAAAGCGGACTCATTCTTCCTGTTTGATTTTCGCCTCTTTTTCC
F12	*SHS1* UTR +30 F	TATAATTGCTAGAAAAATATATTATTAATCATGAGCACTGCTTCAAC
R9	*SHS1* UTR -30 R	GCTCAGCTTTGGATTTTGTACAGATACAACACTGGATGGCGGCGTTAGTATCGAATC
F13	*HIS3* UTR +30 F	AATGAGCAGGCAAGATAAACGAAGGCAAAG
R10	*HIS3* UTR -30 R	GTATGCTGCAGCTTTAAATAATCGGTGTCA
F14	Int Kan F	CGGTTGCATTCGATTCCTGTTTGTAATTGTCC
R11	*SHS1* UTR -469 R	CCACTAGAGCATTGGCAGAATTATATGGTAGT

[1]UTR +/- designates sequence upstream (5') or downstream (3') of the specified gene.

[2]"Int" designates a position internal to the coding sequence of the specified ORF.
