# Supplementary material for: mCAL: A New Approach for Versatile Multiplex Action of Cas9 Using One sgRNA and Loci Flanked by a Programmed Target Sequence
Source: G3 (Bethesda). 2016 May 13;6(7):2147–56. doi: 10.1534/g3.116.029801 (PMC4938667; doi:10.1534/g3.116.029801)
Supplement: Supplemental Material [file supp_g3.116.029801_FileS1.pdf]

SUPPLEMENTAL REFERENCES

Aitchison, J. D., M. P. Rout, M. Marelli, G. Blobel and R. W. Wozniak, 1995 Two novel related yeast nucleoporins Nup170p and Nup157p: complementation with the vertebrate homologue Nup155p and functional interactions with the yeast nuclear pore-membrane protein Pom152p. J Cell Biol 131**:** 1133-1148.

Amberg, D. C., D. J. Burke and J. N. Strathern, 2006 Yeast DNA isolation: miniprep. CSH Protoc 2006.

Boeke, J. D., F. LaCroute and G. R. Fink, 1984 A positive selection for mutants lacking orotidine-5'-phosphate decarboxylase activity in yeast: 5-fluoro-orotic acid resistance. Mol Gen Genet 197**:** 345-346.

Brachmann, C. B., A. Davies, G. J. Cost, E. Caputo, J. Li *et al.*, 1998 Designer deletion strains derived from *Saccharomyces cerevisiae* S288C: a useful set of strains and plasmids for PCR-mediated gene disruption and other applications. Yeast 14**:** 115-132.

Braglia, P., R. Percudani and G. Dieci, 2005 Sequence context effects on oligo(dT) termination signal recognition by *Saccharomyces cerevisiae* RNA polymerase III. J Biol Chem 280**:** 19551-19562.

Christianson, T. W., R. S. Sikorski, M. Dante, J. H. Shero and P. Hieter, 1992 Multifunctional yeast high-copy-number shuttle vectors. Gene 110**:** 119-122.

DiCarlo, J. E., J. E. Norville, P. Mali, X. Rios, J. Aach *et al.*, 2013 Genome engineering in *Saccharomyces cerevisiae* using CRISPR-Cas systems. Nucleic Acids Res 41**:** 4336-4343.

Eckert-Boulet, N., M. L. Pedersen, B. O. Krogh and M. Lisby, 2012 Optimization of ordered plasmid assembly by gap repair in *Saccharomyces cerevisiae*. Yeast 29**:** 323-334.

Fabre, E., and E. Hurt, 1997 Yeast genetics to dissect the nuclear pore complex and nucleocytoplasmic trafficking. Annu Rev Genet 31**:** 277-313.

Finnigan, G. C., and J. Thorner, 2015 Complex in vivo ligation using homologous recombination and high-efficiency plasmid rescue from *Saccharomyces cerevisiae*. Bio-protocol 5**:** e1521.1-e1521.14.

Goldstein, A. L., and J. H. McCusker, 1999 Three new dominant drug resistance cassettes for gene disruption in *Saccharomyces cerevisiae*. Yeast 15**:** 1541-1553.

Horwitz, A.A., J.M. Walter, M.G. Schubert, S.H. Kung, K. Hawkins *et al.*, 2015 Efficient multiplexed integration of synergistic alleles and metabolic pathways in yeasts via CRISPR-Cas. Cell Systems 1**:** 88-96.

Jiang, W., D. Bikard, D. Cox, F. Zhang and L. A. Marraffini, 2013 RNA-guided editing of bacterial genomes using CRISPR-Cas systems. Nat Biotechnol 31**:** 233-239.

Jinek, M., K. Chylinski, I. Fonfara, M. Hauer, J. A. Doudna *et al.*, 2012 A programmable dual-RNA-guided DNA endonuclease in adaptive bacterial immunity. Science 337**:** 816-821.

Kalderon, D., B. L. Roberts, W. D. Richardson and A. E. Smith, 1984 A short amino acid sequence able to specify nuclear location. Cell 39**:** 499-509.

Sikorski, R. S., and P. Hieter, 1989 A system of shuttle vectors and yeast host strains designed for efficient manipulation of DNA in *Saccharomyces cerevisiae*. Genetics 122**:** 19-27.

Versele, M., B. Gullbrand, M. J. Shulewitz, V. J. Cid, S. Bahmanyar *et al.*, 2004 Protein-protein interactions governing septin heteropentamer assembly and septin filament organization in *Saccharomyces cerevisiae*. Mol Biol Cell 15**:** 4568-4583.

Wang, Q., and L. Wang, 2008 New methods enabling efficient incorporation of unnatural amino acids in yeast. J Am Chem Soc 130**:** 6066-6067.

Zheng, L., U. Baumann and J. L. Reymond, 2004 An efficient one-step site-directed and site-saturation mutagenesis protocol. Nucleic Acids Res 32**:** e115.
